# Hypoxia-induced RHCG as a key regulator in psoriasis and its modulation by secukinumab

**DOI:** 10.1063/5.0250742

**Published:** 2025-05-09

**Authors:** Shun Guo, Qian Zhang, Yuan-jie Liu, Yun-yao Hu, Cong Liu, Hui Shen, Jia Liu

**Affiliations:** 1 Department of Dermatology, Affiliated Hospital of Nanjing University of Chinese Medicine, Jiangsu Province Hospital of Chinese Medicine, Nanjing, Jiangsu 210029, People's Republic of China; 2 Department of Dermatology, Zhangjiagang TCM Hospital Affiliated to Nanjing University of Chinese Medicine, Zhangjiagang, 215600 Jiangsu, People's Republic of China; 3 Department of Oncology, Affiliated Hospital of Nanjing University of Chinese Medicine, Jiangsu Province Hospital of Chinese Medicine, Nanjing, Jiangsu 210029, China; 4 No. 1 Clinical Medical College, Nanjing University of Chinese Medicine, Nanjing, Jiangsu 210023, China

## Abstract

The interaction between keratinocytes (KCs) and immune cells is essential in the pathogenesis of psoriasis. Understanding this crosstalk is crucial for developing effective treatment strategies. Recent studies indicate that Rh family C-type glycoprotein (RHCG) enhances cell proliferation and alters cell differentiation; however, its exact pathogenic mechanisms in psoriasis remain unclear. We employed bioinformatics approaches, including spatial transcriptomics analysis, single-cell transcriptomics analysis, and bulk data analysis, to elucidate the biological functions of RHCG. These predictions were validated through *ex vivo* experiments and analysis of clinical specimens. In psoriatic skin, RHCG protein levels were significantly upregulated, with an expanded expression area. Notably, RHCG expression was induced under hypoxic conditions. Furthermore, the upregulation of RHCG enhanced the expression of KC markers S100 Calcium Binding Protein A family (S100A) and Keratin 17 (KRT17), while decreasing Keratin 1 (KRT1) expression. Additionally, RHCG overexpression increased the secretion of C-X-C motif chemokine ligand 14 (CXCL14) from KCs, which subsequently activated dendritic cells. Importantly, treatment with secukinumab effectively ameliorated psoriasis by downregulating RHCG expression and inhibiting associated signaling pathways, whereas glucocorticoid and methotrexate treatments resulted in elevated RHCG expression. These findings indicate that RHCG plays a significant role in hypoxia-induced cellular crosstalk and suggest that RHCG-associated signaling may contribute to the superior efficacy of biological agents compared to conventional hormonal and immunosuppressive therapies.

## INTRODUCTION

Psoriasis is a chronic inflammatory skin disorder characterized by abnormal differentiation of keratinocytes, the primary cellular constituents of the epidermis.^1^ In psoriatic plaques, keratinocytes exhibit rapid proliferation and abnormal differentiation, leading to the development of thickened, scaly lesions.^2^ This dysregulated differentiation manifests as hyperkeratinization and parakeratosis, where keratinocytes retain their nuclei within the stratum corneum, contributing to the distinctive appearance of psoriatic skin.^3^

In addition to aberrant keratinocyte differentiation, the release of inflammatory cytokines plays a central role in the pathogenesis of psoriasis.^4^ Keratinocytes, along with immune cells such as T cells, dendritic cells (DCs), and macrophages, secrete a variety of cytokines and chemokines that perpetuate the inflammatory cascade.^5,6^ Key cytokines, including tumor necrosis factor-α (TNF-α), interleukin 17A (IL-17), and interleukin 22 (IL-22), stimulate keratinocyte proliferation and activation, further amplifying the inflammatory response and promoting lesion formation.^7^ Moreover, hypoxic conditions in psoriatic skin, exacerbated by increased thickness and reduced blood flow, intensify inflammation by inducing the expression of hypoxia-inducible factors (HIFs) and fostering the release of additional inflammatory mediators.^8,9^

The intricate communication between immune cells and keratinocytes in psoriatic skin is vital for disease progression.^10^ Immune cell infiltration into the dermis and epidermis facilitates interactions with keratinocytes through cytokine and chemokine signaling pathways.^11^ This crosstalk triggers the activation of inflammatory pathways, perpetuating the formation of psoriatic plaques.

Recent advancements in biologic therapies have emerged as promising treatment options for psoriasis, offering significant advantages over traditional therapies such as corticosteroids and immunosuppressants.^12,13^ Biologics target-specific cytokines or cell surface receptors implicated in the inflammatory pathways of psoriasis, providing a more focused and effective treatment approach with fewer systemic side effects.^14,15^ The success of biologics underscores the importance of understanding the complex interplay between keratinocyte differentiation, inflammatory cytokine release, immune cell communication, and hypoxia, highlighting the potential for developing novel therapeutic strategies to alleviate symptoms and slow the progression of this chronic skin disease.^16,17^

Emerging research has begun to investigate the role of Rh family C-type glycoprotein (RHCG) in psoriasis pathogenesis.^18,19^ Preliminary studies suggest that alterations in RHCG expression or function may contribute to the abnormal proliferation and differentiation of keratinocytes.^19^ While the direct involvement of RHCG in inflammatory pathways has not been definitively established, emerging evidence indicates a potential connection. For example, RHCG may influence the skin's pH microenvironment,^20^ modulating the activity of immune cells and cytokines involved in the inflammatory cascade. Additionally, RHCG could regulate ammonium levels,^21^ impacting the metabolic state of keratinocytes and immune cells within the skin. Although these findings are promising, further investigation is necessary to clarify the role of RHCG in psoriasis and explore its potential as a therapeutic target.

This study aims to investigate the associations between RHCG and cellular crosstalk in the context of psoriasis, with the ultimate goal of elucidating the underlying molecular mechanisms and assessing the feasibility of targeting RHCG therapeutically. Our results provide insights into the role of RHCG in regulating hypoxia-related pathways and offer novel perspectives on the mechanisms underlying the superior efficacy of biological treatments in psoriasis.

## RESULTS

### RHCG expression level and area are upregulated in psoriasis lesions

**FIG. 1.**
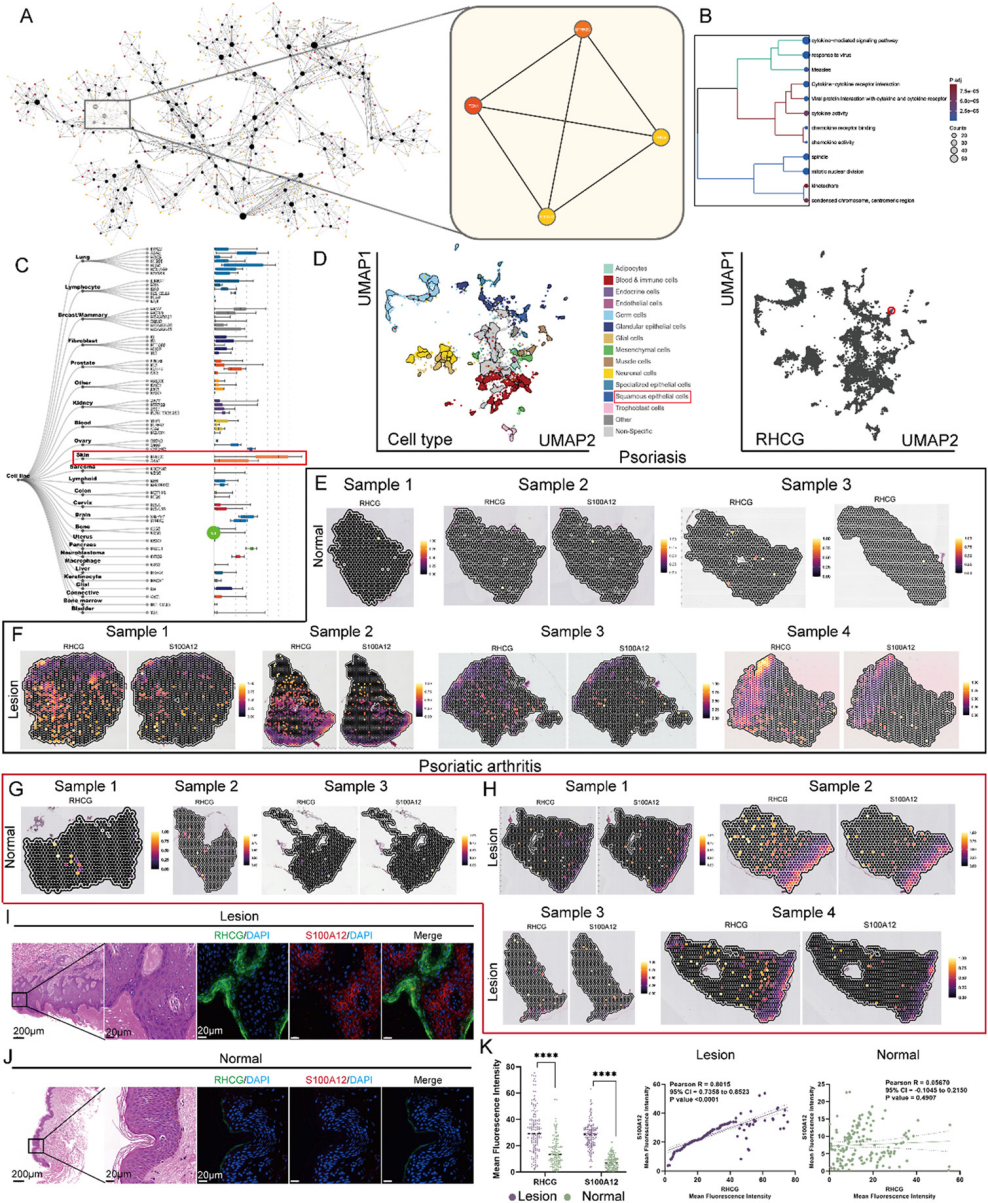
Bulk data combined with spatial transcriptomics (ST) to characterize RHCG expression characteristics. (a) The Multiscale Embedded Gene Co-Expression Network Analysis (MEGENA) network shows the gene module we are concerned about. The more important the gene in the module, the darker its color. Since we have previously identified RHCG as a psoriasis-associated pathogenic factor, the RHCG-associated subnetwork was extracted (displayed in the right part of the graph). *TCN1*, *SPRR2C*, and *S100A12* are thought to be highly co-expressed with *RHCG*. Due to the lack of reports on the association between TCN1/SPRR2C and psoriasis, *S100A12* was chosen for subsequent RHCG-related studies. (b) Gene ontology (GO) and Kyoto Encyclopedia of Genes and Genomes (KEGG) enrichment analysis of *RHCG*-related genes (the top 50 most relevant genes were used). The larger the circle and the redder the color, the more significant the enriched term. (c) Expression of *RHCG* in various cell lines from different tissues. Skin-derived cells had the highest RHCG expression compared to cells from other tissues. (d) In single-cell transcriptome data across diverse tissues, *RHCG* is identified to be a part of cluster 59 (suprabasal KCs, red circle). Spatial feature plots of the expression of *RHCG* and *S100A12* in tissue sections of psoriatic skin (e) and paired normal skin (f). Data were obtained from the CROST portal, and a total of four psoriasis skin specimens (samples 1–4) and four normal controls (samples 1–4) were included in this study. If a gene is not expressed in the sample, the corresponding image is omitted. Psoriasis samples 1–4 and corresponding normal samples 1–4 were used in this study. See also Fig. S2. Spatial feature plots of the expression of RHCG and S100A12 in tissue sections of psoriatic arthritis skin (g) and paired normal skin (h). Data were obtained from the CROST portal, and a total of four psoriatic arthritis skin specimens (samples 1–4) and four normal controls (samples 1–4) were included in this study. If a gene is not expressed in the sample, the corresponding image is omitted. Psoriatic arthritis samples 1–4 and corresponding normal samples 1–4 were used in this study. See also Fig. S3. Representative mIF staining of human psoriasis (i) and normal (j) skin tissues. RHCG (green), S100A12 (red), and DAPI (blue) in individual and merged channels are shown. Scale bars, 200 *μ*m (panoramic view) and 20 *μ*m (zoomed view). The experiment was performed on 30 independent patients (30 psoriasis samples and 30 adjacent normal controls). (k) Wilcoxon test was used to compare the differences in RHCG and S100A12 expression between psoriasis and normal skin (left), and Spearman correlation was used to measure the correlation between RHCG and S100012 in different tissues (right). Not only are both RHCG and S100A12 highly expressed in psoriatic skin (P < 0.0001), but both are highly co-localized in psoriasis (R = 0.8015). For each specimen, five random fields of view were randomly selected and captured. ^****^P < 0.0001.

We obtained bulk RNA sequencing data on psoriasis and associated co-expression networks from previous studies (Fig. S1). Focusing on the role of RHCG in psoriasis, we extracted the C1_2 subnetwork, which includes RHCG. Notably, we observed a strong correlation between RHCG and S100 Calcium Binding Protein A12 (S100A12) [Fig. 1(a)]. Like other calcium-binding proteins, S100A12 is upregulated in inflammatory conditions and is believed to play a significant role in the immunopathology of psoriasis.^22^

To elucidate the signaling pathways associated with RHCG, we conducted a functional enrichment analysis on the C1_2 subnetwork. As shown in Fig. 1(b), RHCG may play an important role in cytokine activity-related pathways. Additionally, RHCG demonstrated a distinct skin-biased expression [Fig. 1(c)], further supported by scRNA-seq data across various cell types, particularly squamous epithelial cells [Fig. 1(d)].

The spatial distribution of genes within tissues often correlates closely with their functional attributes. To further investigate the potential role of RHCG in psoriasis development, we performed ST RNA-seq analysis to characterize the spatial patterns of *RHCG* expression. Utilizing the built-in annotation information from CROST, we classified the spots into composite cell types, including differentiated keratinocytes (KCs), fibroblasts, migratory dendritic cells, pericytes, Schwann cells, T cells, and vascular endothelium (Figs. S2 and S3 for psoriasis tissue and matched normal tissue, as well as psoriatic arthritis tissue and matched normal tissue). The spatial distribution of *RHCG* was depicted, revealing that in psoriasis and psoriatic arthritis, RHCG expression encompasses a larger area in lesional tissues compared to normal tissues [Figs. 1(e)–1(h)]. To investigate whether RHCG could serve as a circulating biomarker for psoriasis, we analyzed transcriptomic data from the GSE55201 dataset comparing RHCG expression levels in peripheral blood between psoriasis patients and healthy volunteers. Interestingly, despite the prominent upregulation observed in skin tissue, analysis revealed no significant differences in RHCG transcript levels in peripheral blood between these groups (Fig. S4A). To further validate these findings, we collected serum samples from 30 psoriasis patients and 30 healthy controls for protein analysis. Consistent with the transcriptomic data, enzyme-linked immunosorbent assay (ELISA) measurements demonstrated that RHCG protein levels in serum were relatively low in both groups, with no statistically significant differences observed (Fig. S4B). These results suggest that while RHCG plays an important role locally in psoriatic skin, its expression may not extend systemically at detectable levels, limiting its potential utility as a serum biomarker for monitoring disease activity.

For experimental validation, we performed multiple fluorescence assays along with corresponding H&E staining on paraffin sections of psoriasis and normal skin. The images of RHCG and S100A12 staining demonstrated high expression levels and co-localization in psoriatic tissues [Figs. 1(i)–1(k)], a phenomenon not observed in normal skin.

### RHCG may be associated with aberrant KC differentiation in psoriasis

**FIG. 2.**
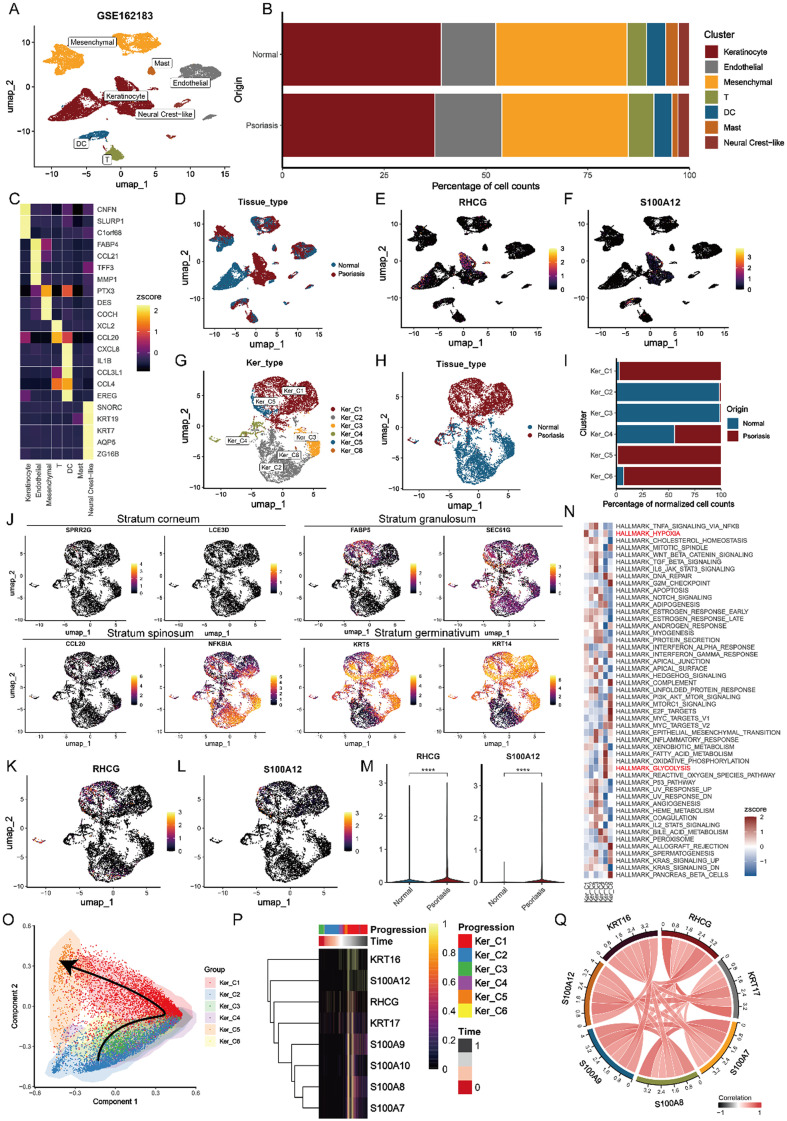
Identification of potential functions of RHCG in psoriasis by single-cell transcriptome. (a) Uniform manifold approximation and projection (UMAP) plot showing the transcriptome landscape of GSE162183, including three psoriasis skins and three normal controls). Twenty-two cell clusters were annotated as seven major cell types. Mesenchymal cells, mast cells, endothelial cells, keratinocytes, neural creat-like cells, DC, and T cells. (b) The clustering of cellular components and their respective composition proportions in both normal and psoriatic skin samples was examined, with the cellular types serving as the basis for color coding in the analysis. (c) Heatmap showing the percentage of expressed cells and average expression levels of well-known marker genes of the seven cell types. (d) UMAP plot of the classification of tissue origins. UMAP plots of the expression of *RHCG* (e) and *S100A12* (f). Both are expressed restrictively in KCs. UMAP plot of all KCs color-coded for keratinocyte type (Ker_type, g) and tissue type (h). (i) The proportion of different tissue types in six Ker_types. (j) UMAP plots of the expression of marker genes of KCs in the stratum corneum (*SPRR2G* and *LCE3D*), the stratum granulosum (*FABP5* and *SEC61G*), the stratum spinosum (*CCL20* and *NFKBIA*), and the stratum basale (*KRT14* and *KRT5*). UMAP plots of the expression of *RHCG* (k) and *S100A12* (l) in KCs. Both are highly expressed in the stratum granulosum. (m) Violin plots showing the differential expression of *RHCG* and *S100A12* between psoriasis skins and normal controls (unpaired Wilcoxon test). (n) Heatmap showing the activity of hallmark gene sets in different Ker_types. Hypoxia and glycolysis are highlighted (red) for their important role in psoriasis. (o) Pseudotime analysis for the inferred trajectory of KCs from Ker_C2 to Ker_C5. (p) KCs are ordered along the linear trajectories inferred from Ker_types. Time represents the inferred time point from the pseudotime also shown with the molecular annotations. We focus on RHCG and genes that are known to be associated with an aberrant differentiation state of KCs. (q) The Spearman correlation between RHCG and the gene of interest in the bulk data. ^****^P < 0.0001.

To further elucidate the expression of RHCG and its role in the pathology of psoriasis, we analyzed a scRNA-seq cohort from a previous study, which included samples from three lesional psoriatic tissues and three matched non-lesional (normal) skin tissues. Utilizing the knowledge-based approach within the Deeply Integrated human Single-Cell Omics (DISCO) database, we identified seven major cellular components: mesenchymal cells, mast cells, endothelial cells, keratinocytes, neural crest-like cells, dendritic cells (DCs), and T cells [Fig. 2(a)]. The heterogeneous cellular proportions in each tissue type are depicted in Fig. 2(b), with well-established cellular markers presented in a heatmap [Fig. 2(c)]. Our analysis revealed that KCs in lesional tissues exhibited the highest expression levels of *RHCG* and *S100A12* [Figs. 2(d)–2(f)]. To further investigate the heterogeneity of RHCG expression, we performed a clustering analysis of all KCs and identified six prominent subpopulations [Ker_C1-C6, Figs. 2(g) and 2(h)]. The proportions of Ker_C1-C6 varied significantly between the two tissue types [Fig. 2(i)]. Specifically, Ker_C1, Ker_C5, and Ker_C6 were predominantly derived from lesion samples [Fig. 2(i)]. Interestingly, keratinocytes with high *RHCG* and *S100A12* expression originated primarily from the stratum granulosum, characterized by markers such as *FABP5* and *SEC61G* [Figs. 2(j)–2(l)]. Both *RHCG* and *S100A12* were significantly upregulated in lesion tissues compared to normal tissues (P < 0.0001) [Fig. 2(m)], which was also supported by corresponding protein-level data. In addition, enrichment analyses based on Hallmark gene sets highlighted that the hypoxia pathway (marked in red) was the most prominent feature of Ker_C1 [Fig. 2(n)]. To gain insights into the dynamic evolution of keratinocytes from normal to lesional states, we utilized the Slingshot tool for trajectory inference [Fig. 2(o)]. Our findings revealed that consistent with known markers of abnormal KC differentiation (such as *KRT17*) and several pro-inflammatory cytokines, *RHCG* expression was predominantly observed during intermediate and advanced stages of differentiation, corresponding to lesional stages [Fig. 2(p)]. Furthermore, results from bulk sequencing data supported a positive correlation between *RHCG* and these pathogenic factors associated with psoriasis [Fig. 2(q)]. Taken together, these findings suggest that RHCG may have a pathogenic role in psoriasis, particularly in the context of aberrant KC differentiation.

### Hypoxia induces RHCG expression

The epidermis, the outermost layer of the skin, primarily comprises KCs undergoing successive maturation and differentiation, along with a small proportion of other cell types. This layer is relatively thin and lacks a direct blood vessel supply of oxygen, instead relying on oxygen diffusion from the dermis.^23^ It has been reported that oxygen-dependent hypoxia-inducible factor (HIF) isoforms are significantly upregulated in psoriatic lesions and patient serum,^24^ contributing to abnormal vascularization and inflammation.^25,26^ Thus, hypoxia-related pathways are likely involved in the pathogenesis of psoriasis.

**FIG. 3.**
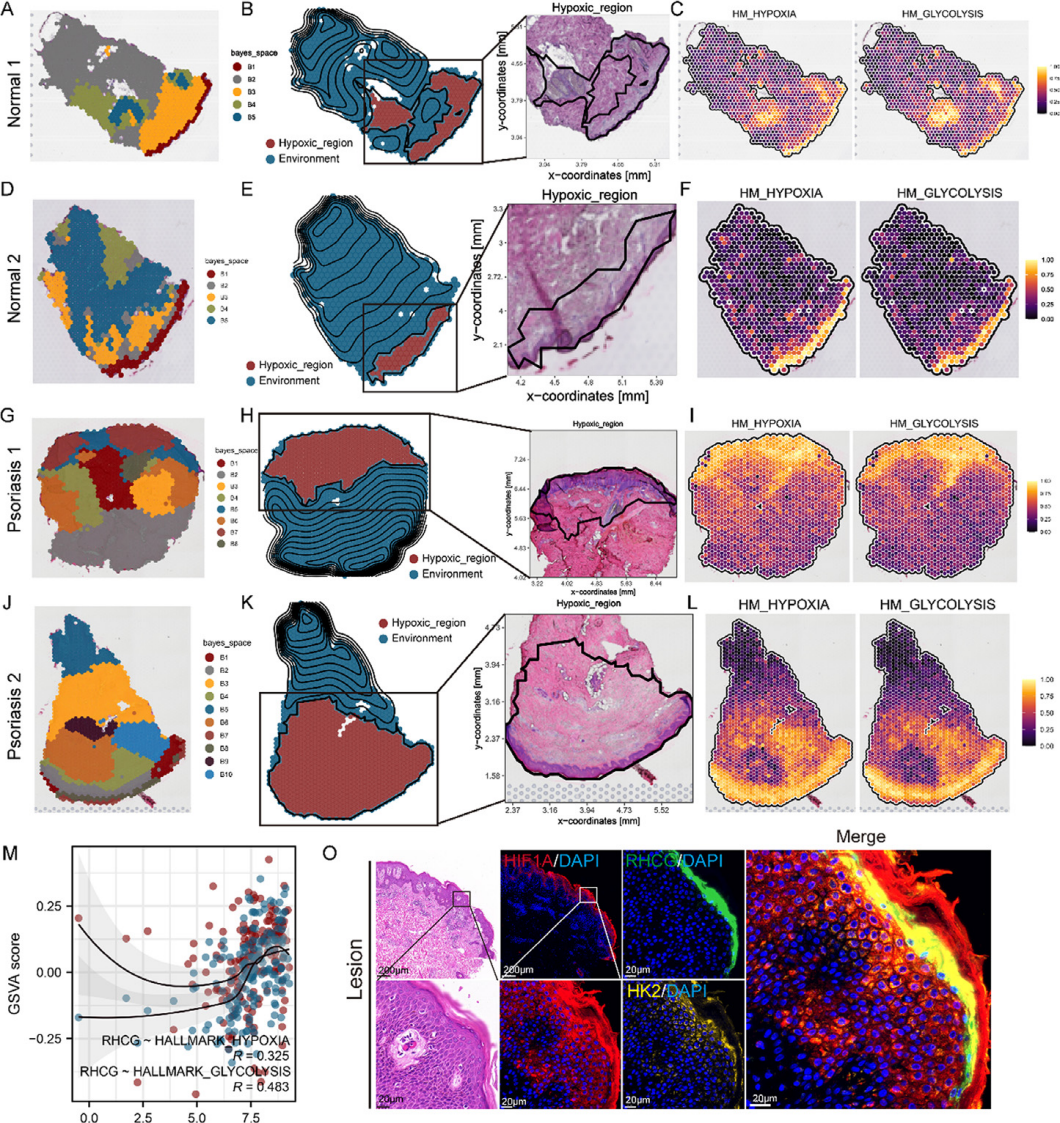

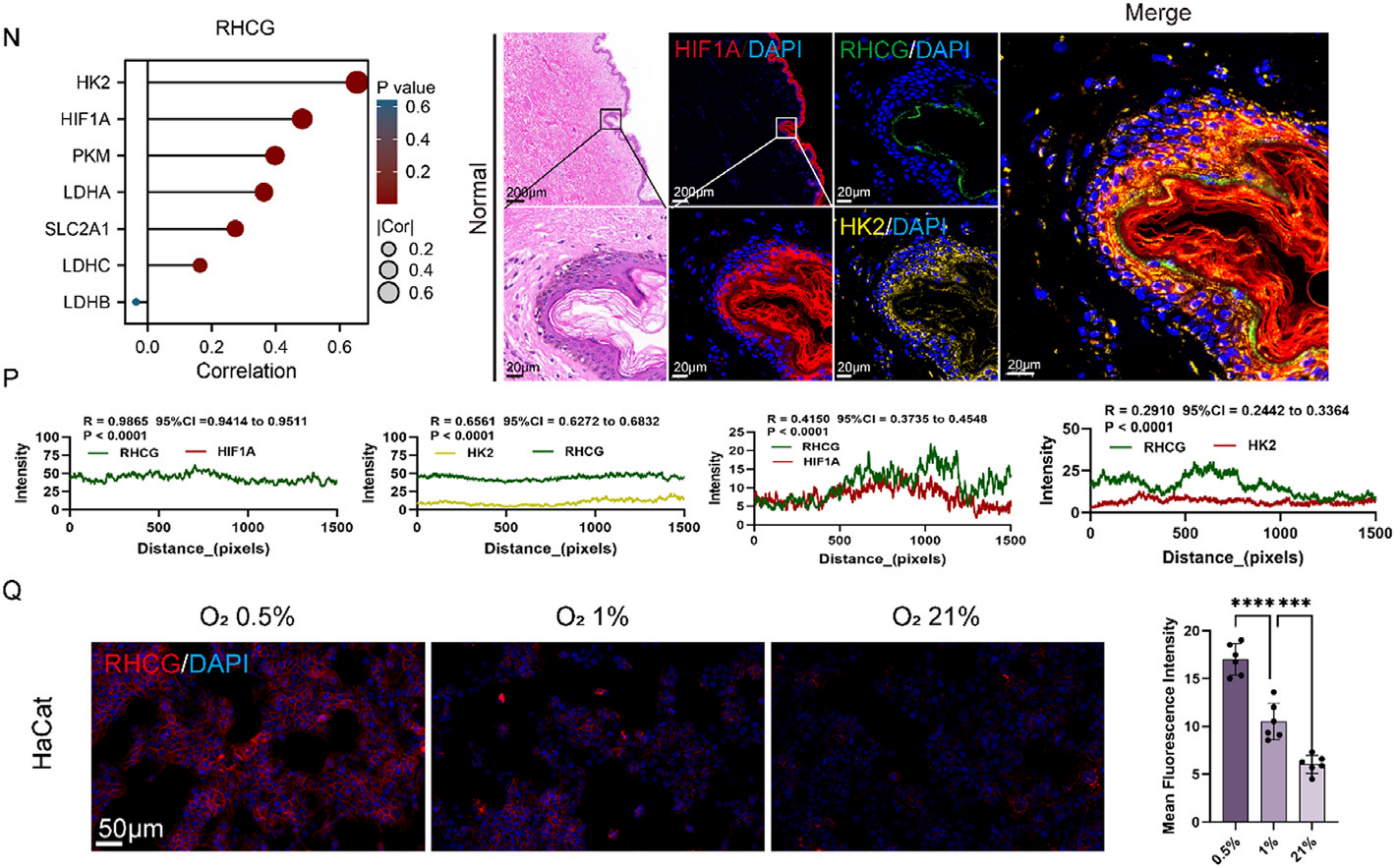
Spatial transcriptome combined with *in vitro* experiments to determine the relationship between RHCG and hypoxia as well as glycolysis. Unbiased clustering of ST spots based on the BayesSpace method in Normal sample 1 (a), Normal sample 2 (d), Psoriasis sample 1 (g), and Psoriasis sample 2 (j). See also Figs. S4A, 4D, 4G, and 4J. ST spots are annotated based on numeric variables of hypoxia (GSVA score) automatically. An area of interest named hypoxic region is highlighted in Normal sample 1 (b), Normal sample 2 (e), Psoriasis sample 1 (h), and Psoriasis sample 2 (k). See also Figs. S4B, 4E, 4H, and 4K. Spatial feature plots of the signature score of hypoxia and glycolysis (GSVA score) in Normal sample 1 (c), Normal sample 2 (f), Psoriasis sample 1 (i), and Psoriasis sample 2 (l). See also Figs. S4C, 4F, 4I, and 4L. (m) The Spearman correlations between *RHCG* and the Geneset variation analysis (GSVA) score of hypoxia and glycolysis in the bulk data. (n) The Spearman correlations between *RHCG* and the GSVA score of key glycolytic enzyme-coding genes in the bulk data. *HK2* showed the strongest correlation with *RHCG*. (o) Representative mIF and HE staining of human psoriasis and normal skin tissue. HIF1A (red), RHCG (green), HK2 (gold), and DAPI (blue) in individual and merged channels are shown. Scale bars, 200 *μ*m (panoramic view) and 20 *μ*m (zoomed view). To highlight the expanded hypoxic area of lesion tissue compared to normal controls, both panoramic and localized views of HIF1A are provided. The experiment was performed on 30 independent patients (30 psoriasis samples and 30 adjacent normal controls). (p) Spatial intensity correlations between *RHCG* and *HIF1A* as well as *HK2*. The graphs represent the correlation (Spearman method) of fluorescence intensity across distances in the lesion tissues and normal controls. Stronger positive correlations are observed between *RHCG* and *HIF1A* (R = 0.9865, P < 0.0001) as well as *HK2* (R = 0.6561, P < 0.0001) in lesion tissues than in normal controls. (q) Effects of hypoxia (Tri-Gas Incubator of different concentrations of oxygen) on RHCG expression in the HaCaT cell line. Experiments were carried out in triplicate, and the resulting data were presented as mean ± SD. ns represented no significance; ^***^P < 0.001, ^****^P < 0.0001, by ANOVA.

To investigate this, we analyzed ST data from four normal skin samples and four psoriatic skin samples. We observed increased hypoxic regions in the lesional tissues compared to the normal controls [Figs. 3(a)–3(l) and Fig. S5]. Notably, a concurrent increase in glycolytic activity was also evident in the lesional tissues [Figs. 3(c), 3(f), 3(i), and 3(l)]. Emerging research suggests that HIF-1α, activated under hypoxic conditions, plays a key role in enhancing glycolysis by upregulating key glycolytic enzymes and glucose transporters, such as glucose transporter 1 (GLUT1) and hexokinase 2 (HK2), in KCs.^27,28^ This enhanced glycolytic activity may contribute to the sustained proliferation and aberrant differentiation of KCs observed in psoriasis.

Our bulk analysis further corroborated these findings, revealing a positive correlation between *RHCG* expression, hypoxia (R = 0.325), and glycolysis (R = 0.483) [Fig. 3(m)]. These observations led us to hypothesize that overexpression of RHCG may be associated with hypoxia and glycolysis in psoriasis. To explore the possible mechanisms, we calculated the correlation between *RHCG* and genes encoding seven key enzymes involved in glycolysis at the transcriptional level [Fig. 3(n)]. Our results showed that *HK2* was significantly and positively correlated with *RHCG* (R > 0.6).

To further investigate the relationship among HIF-1α, HK2, and RHCG in psoriatic skin, we performed multiplex immunofluorescence labeling, which demonstrated stronger co-localization of these three components in lesional tissues compared to healthy tissues [Figs. 3(o) and 3(p)]. This suggests a close association of RHCG with hypoxia-related signaling pathways in psoriasis.

To directly test whether hypoxia activates RHCG expression, we exposed HaCaT cells to various concentrations of ambient oxygen for 24 h. As expected, RHCG protein staining was intensified in HaCaT cells under hypoxic conditions [Fig. 3(q)]. These results indicate that RHCG overexpression in psoriasis may be driven by hypoxia and may thus play a role in downstream metabolic alterations contributing to disease progression.

### Upregulation of RHCG leads to impaired KC differentiation and an inflammatory phenotype

**FIG. 4.**
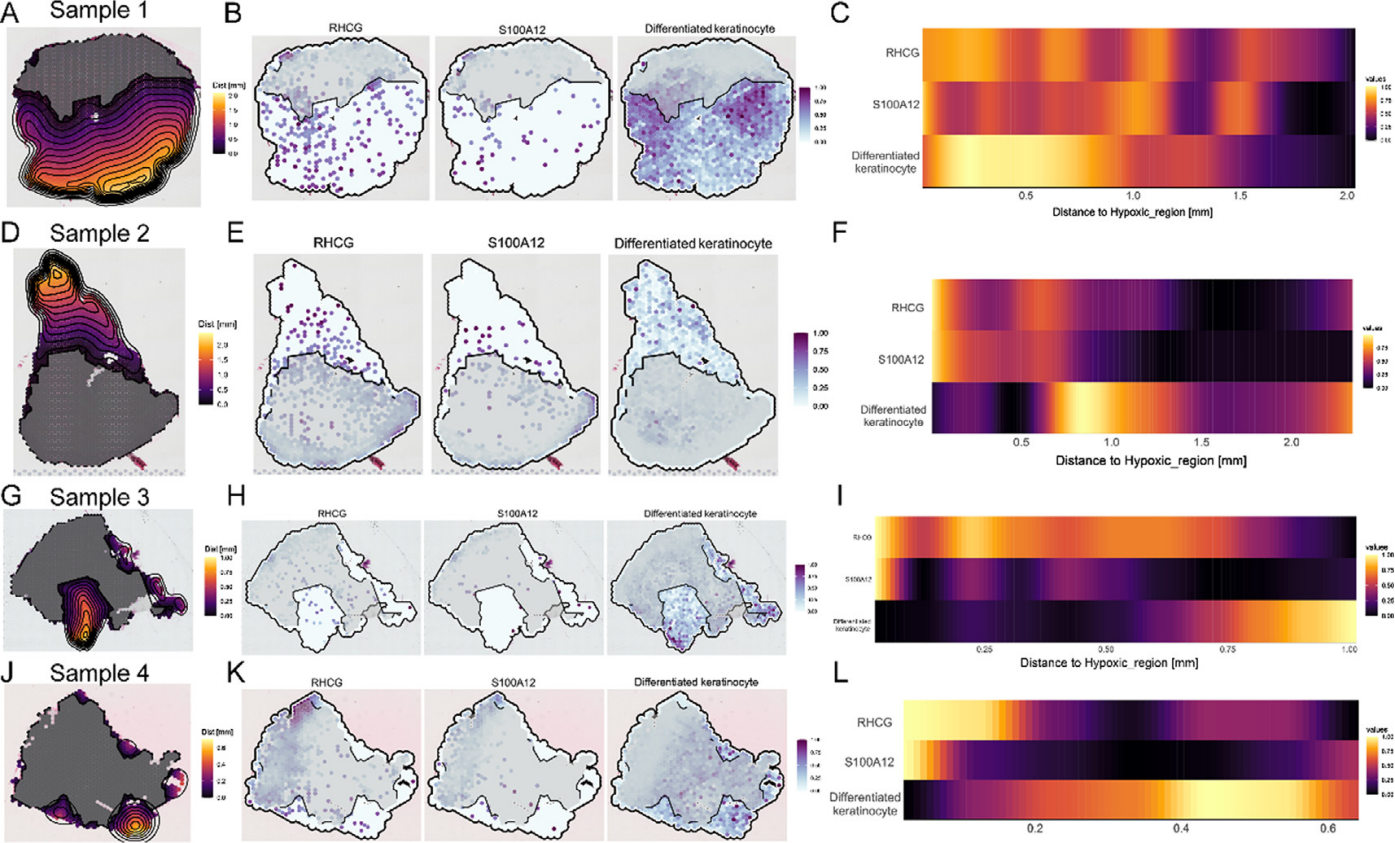

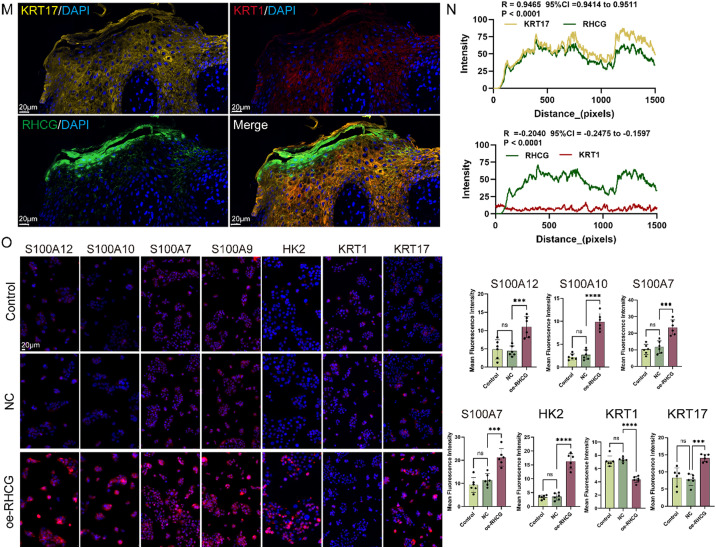
Spatial transcriptome combined with *in vitro* experiments to determine the role of RHCG in the regulation of aberrant differentiation and inflammatory phenotypes of KCs. The borders of hypoxic areas [Figs. 3(a), 3(d), 3(g), and 3(j)] are used as reference points to analyze the expression trends of *RHCG*, *S100A12*, and differentiated KCs along the distance to the annotated areas in Psoriasis sample 1 (a), Psoriasis sample 2 (d), Psoriasis sample 3 (g), and Psoriasis sample 4 (j). The farther the distance from the hypoxic area, the brighter the color. Spatial feature plots showing the correlations between the expression of *RHCG*, *S100A12*, and differentiated DCs with the distances to hypoxic regions in Psoriasis sample 1 (b), Psoriasis sample 2 (e), Psoriasis sample 3 (h), and Psoriasis sample 4 (k). The higher the level of expression, the darker the color of the dot. Heatmaps showing the correlations between the expression of *RHCG*, *S100A12*, and differentiated DCs with the distances to hypoxic regions in Psoriasis sample 1 (c), Psoriasis sample 2 (f), Psoriasis sample 3 (i), and Psoriasis sample 4 (l). The higher the level of expression, the darker the color. (m) Representative mIF staining of human psoriasis skin tissue. KRT17 (gold), KRT1 (red), RHCG (green), and DAPI (blue) in individual and merged channels are shown. Scale bars, 20 *μ*m. The experiment was performed on 30 independent patients (30 psoriasis samples and 30 adjacent normal controls). (n) Spatial intensity correlations between *RHCG* and *KRT17* as well as *KRT1*. The graphs represent the correlation (Spearman method) of fluorescence intensity across distances in the lesion tissues. A stronger positive correlation is observed between RHCG and KRT17 (R = 0.9465, P < 0.0001) in lesion tissues. (o) Fluorescence images demonstrating the expression of markers of interest (S100A12, S100A10, S100A7, S100A9, HK2, KRT1, KRT17) in different treatment conditions (control, NC, and oe-RHCG). Significant differences in the mean fluorescence intensity of these markers are noted, reflecting the impact of RHCG overexpression on their expression in the HaCaT cell line. Experiments were carried out in triplicate, and the resulting data were presented as mean ± SD. ns represented no significance; ^***^P < 0.001, ^****^P < 0.0001, by ANOVA.

Psoriasis is characterized by the abnormal differentiation and proliferation of KCs, with hypoxia playing a pivotal role in this process.^29^ Given the positive regulation of RHCG expression under hypoxic conditions, we next sought to investigate the relationship between RHCG and KC differentiation. To this end, we used ST data to analyze nonrandom expression patterns of specific gene features (*RHCG*, *S100A12*, and differentiated KCs) associated with hypoxic characteristics. We first defined the spatial distribution of distances from hypoxic regions [Figs. 4(a), 4(d), 4(g), and 4(j)]. As depicted in Figs. 4(b), 4(c), 4(e), 4(f), 4(h), 4(i), 4(k), and 4(l), we observed varying expression gradients between differentiated KCs and *RHCG*/*S100A12* in three of the four psoriasis samples analyzed. Keratins, as major structural intermediate filament proteins in KCs, display highly specific expression patterns at different stages of KC differentiation.^30^ In normal skin, KRT1 and KRT10 are typically expressed in suprabasal differentiated KCs.^31^ However, in response to skin injury, KCs near the wound downregulate KRT1 and KRT10 while upregulating KRT16 and KRT17.^32^ In the current study, mIF was employed to examine the association between RHCG and keratins, specifically KRT1 and KRT17 [Figs. 4(m) and 4(n)]. The staining results revealed that KRT17 was predominantly expressed in regions of high RHCG expression, whereas KRT1 expression was comparatively weak. These findings suggest that RHCG overexpression is closely linked to aberrant KC phenotypes. It is well-documented that the dysregulated expression of keratins not only alters KC proliferation, migration, and differentiation but also enhances their inflammatory and metabolic features.^33,34^ In our *in vitro* experiments, we found that overexpression of RHCG significantly increased the levels of S100A (an inflammatory marker), HK2 (a key enzyme in glycolysis), and KRT17, while inhibiting the expression of KRT1 [Fig. 4(o)]. These results indicate that RHCG acts as a factor driving KCs toward a pathogenic phenotype characterized by altered differentiation and increased inflammatory and glycolytic activity.

### Upregulation of RHCG enhances the communication between KCs and DCs to promote DC activation

DCs, known for their role in antigen presentation and T cell activation, are profoundly influenced by psoriatic KCs.^35,36^ The interaction between activated KCs and DCs involves a complex network of cytokines and chemokines that amplifies the inflammatory response.^37,38^ For instance, the byproducts of the ornithine cycle in psoriatic KCs, including polyamines, synergize with autoantigens to form potent complexes that activate myeloid DCs,^39^ resulting in the production of multiple inflammatory factors. This cascade further perpetuates inflammation in psoriasis, highlighting the critical role of KCs in promoting DC activation and exacerbating the disease.^40^

**FIG. 5.**
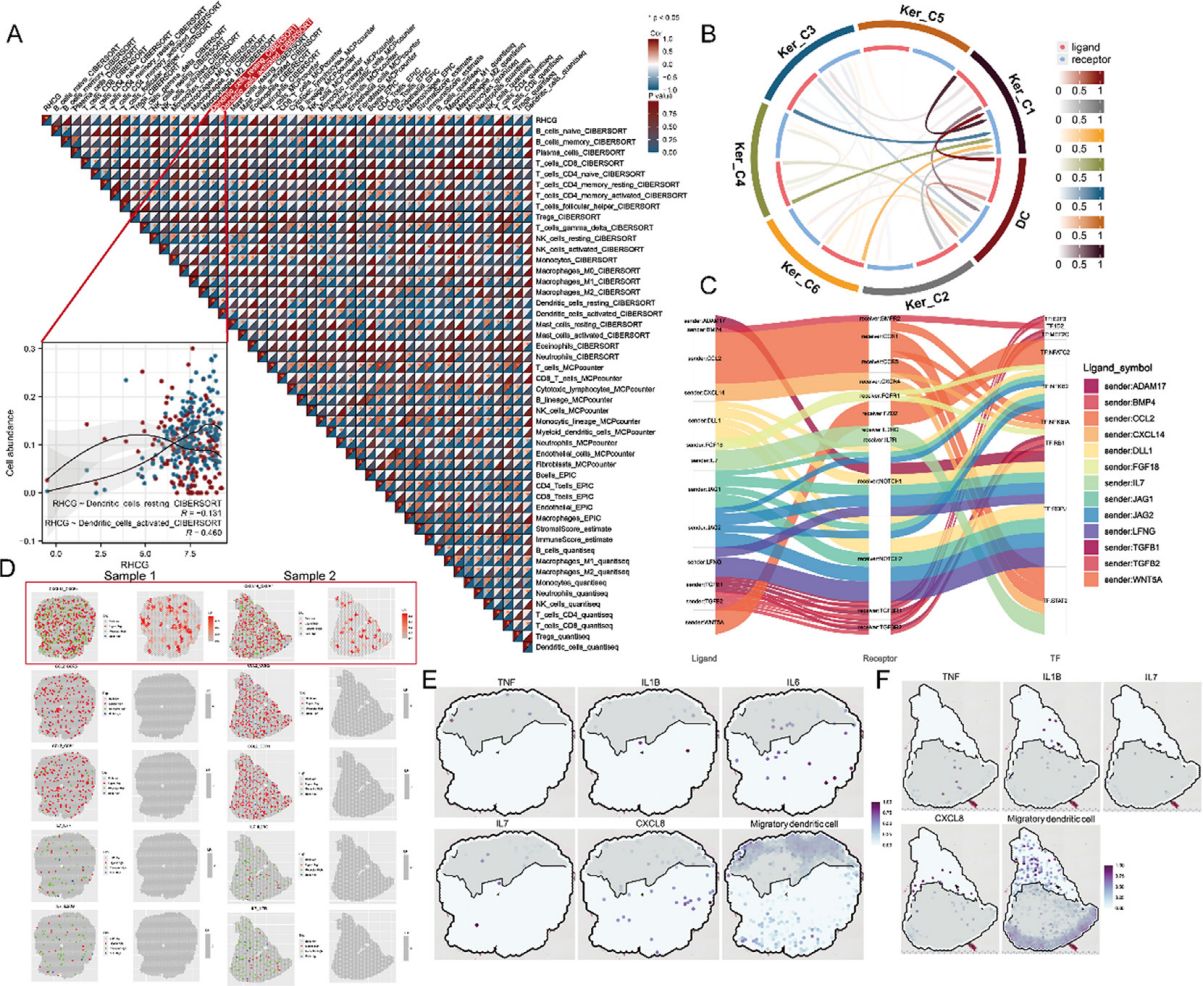

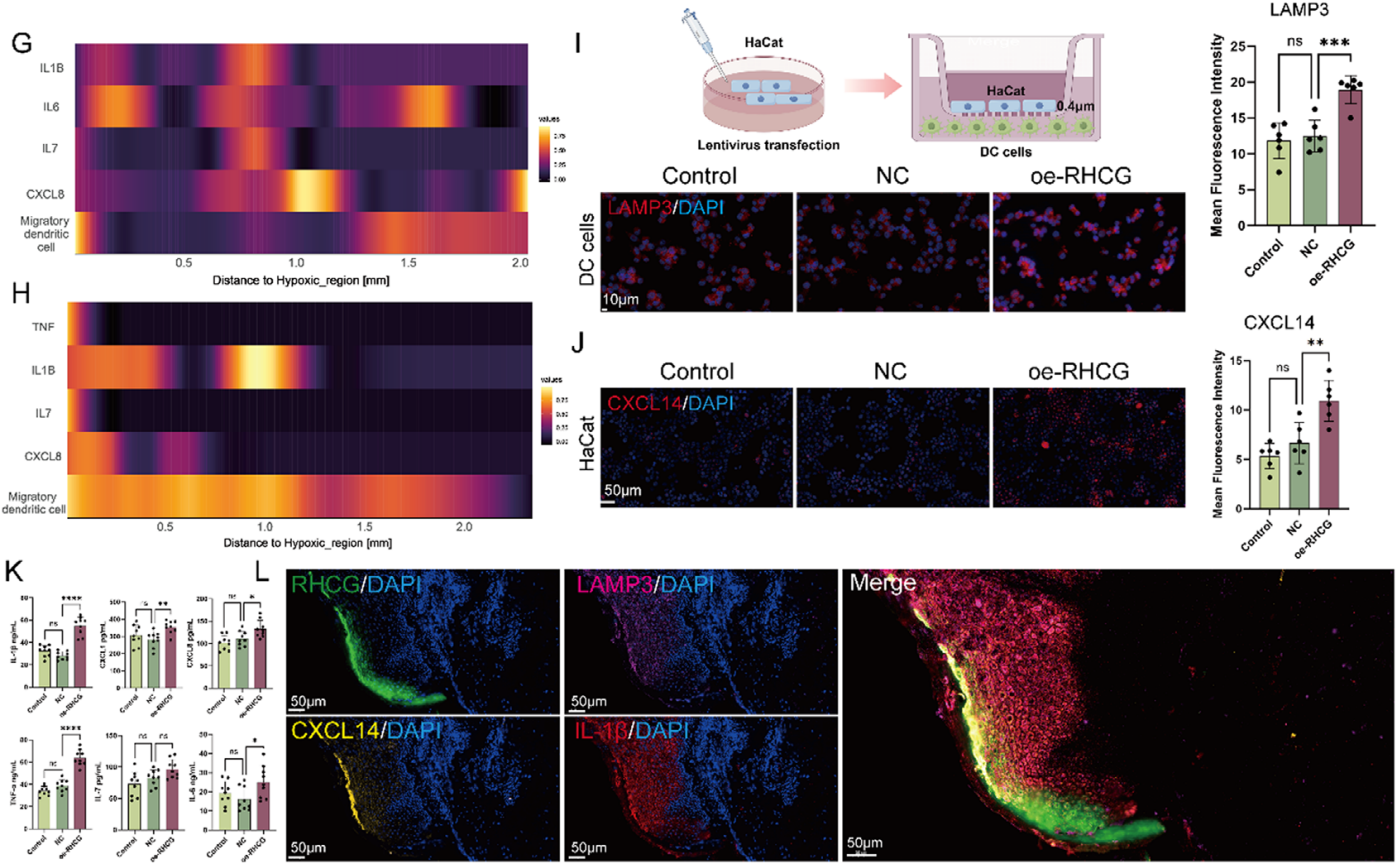
Interaction between KCs and DCs. (a) Correlations between *RHCG* expression and the infiltration level of immune cells. The CIBERSORT, MCPcounter, EPIC, estimate, and quantiseq algorithms were applied for the immune infiltration estimations. We specifically focused on the correlation between RHCG and DCs and provided corresponding scatter plots of the correlation between RHCG and activated DCs (R = 0.460) as well as resting DCs (R = −0.131). (b) The chord plot shows the interactions between KCs and DCs. (c) Sankey plot showing the detailed L–R–TF axis for the communications between KCs and DCs. STAT2-related L–R pairs were selected for further study because of STAT's important role in regulating the signaling pathways that govern DCs' development and function. (d) Spatial feature plots showing the expression of STAT2-related L–R pairs in Psoriasis sample 1 (left) and Psoriasis sample 2 (right). The higher the level of expression, the redder the color of the dot. See also Figs. S5A and 5B. Spatial feature plots showing the correlations between the expression of inflammatory cytokines with the distances to hypoxic regions in Psoriasis sample 1 (e) and Psoriasis sample 2 (f). The higher the level of expression, the darker the color of the dot. See also Figs. S5C and 5D. Heatmaps showing the correlations between the expression of inflammatory cytokines with the distances to hypoxic regions in Psoriasis sample 1 (g) and Psoriasis sample 2 (h). The higher the level of expression, the darker the color. See also Figs. S5E and 5F. (i) Representative fluorescence images show the expression of LAMP3 in DCs co-cultured with HaCaT cells under different treatment conditions (the schematic diagram of the co-culture system is shown in the upper part). LAMP3 is stained in red, while nuclei are visualized with DAPI (blue). Scale bars indicate 10 *μ*m. (j) Representative fluorescence images show the expression of CXCL14 in HaCaT cells under different treatment conditions. CXCL14 is stained in red, while nuclei are visualized with DAPI (blue). Scale bars indicate 10 *μ*m. (k) The concentration of inflammatory cytokines in cell supernatants KCs was measured by enzyme-linked immunosorbent assay (ELISA). (l) Representative mIF staining of human psoriasis skin tissue. RHCG (green), LAMP3 (pink), CXCL14 (gold), IL-1β (red), and DAPI (blue) in individual and merged channels are shown. Scale bars, 50 *μ*m. The experiment was performed on 30 independent patients (30 psoriasis samples and 30 adjacent normal controls). Experiments were carried out in triplicate, and the resulting data were presented as mean ± SD. ns represented as no significance; ^*^P < 0.05; ^**^P < 0.01; ^***^P < 0.001; ^****^P < 0.0001, by ANOVA.

Building on our previous findings of the association between RHCG and DC activation, we further utilized five different algorithms to estimate immune cell components in bulk sequencing samples. As shown in Fig. 5(a), a significant correlation was observed between *RHCG* expression and the levels of various immune cell infiltrates. Notably, using the CIBERSORT algorithm, we found a strong positive correlation between *RHCG* expression and activated DCs (R = 0.460), while no significant correlation was seen with resting DCs (R = −0.131). These findings strongly imply that RHCG may have a stimulatory effect on DC maturation.

To explore this further, we used the Cellcall tool to compare cellular interactions between different KC subpopulations and DCs. The results revealed that the Ker_C1 subpopulation exhibited more frequent communication with DCs than other KC subpopulations [Fig. 5(b)]. We then analyzed ligand–receptor (LR) and transcription factor (TF) interactions between Ker_C1 and DCs. Among the identified transcription factors, STAT2 was of particular interest [Fig. 5(c)]. Recent studies have implicated STAT2 in regulating DC activation,^41^ specifically by enhancing pro-inflammatory cytokine secretion following TLR-4 activation, independent of type I interferons.^41^ This suggests that STAT2 has a direct role in modulating inflammation and the efficiency of antigen cross-presentation by DCs.

Key STAT2-associated ligand–receptor pairs identified included CXCL14–CXCR4, CCL2–CCR5, CCL2–CCR1, IL7–IL7R, and IL7–IL2RG. Notably, CXCL14–CCR4 (highlighted in red) was highly expressed in lesional tissues [Fig. 5(d) and Figs. S6A and 6B]. We also analyzed the expression gradients of DCs and inflammatory factors in relation to hypoxic regions, finding that the expression of DC markers and inflammatory mediators decreased with increasing distance from hypoxic areas [Figs. 5(e)–5(h) and Figs. S6C–6F]. These findings further support a strong association between RHCG expression and DC activation.

For experimental validation, we developed a KC–DC co-culture model to evaluate the effect of RHCG expression on the DC phenotype. As expected, overexpression of RHCG significantly increased the expression of the DC maturation marker LAMP3 [Fig. 5(i)]. Importantly, compared to the control group, RHCG-overexpressing KCs secreted significantly higher levels of CXCL14 [Fig. 5(j)], suggesting activation of CXCL14–CXCR4 signaling. Furthermore, RHCG overexpression elevated the levels of pro-inflammatory cytokines, including IL-1β, CXCL1, CXCL8, TNF-α, IL-6, and IL-7 [Fig. 5(k)]. These results were further confirmed through mIF analysis of tissue samples [Fig. 5(l)]. Collectively, these findings indicate that RHCG overexpression enhances KC–DC communication by activating CXCL14–CCR4 signaling, thereby promoting DC activation.

### Secukinumab but not glucocorticoids and methotrexate downregulate RHCG expression in KCs

**FIG. 6.**
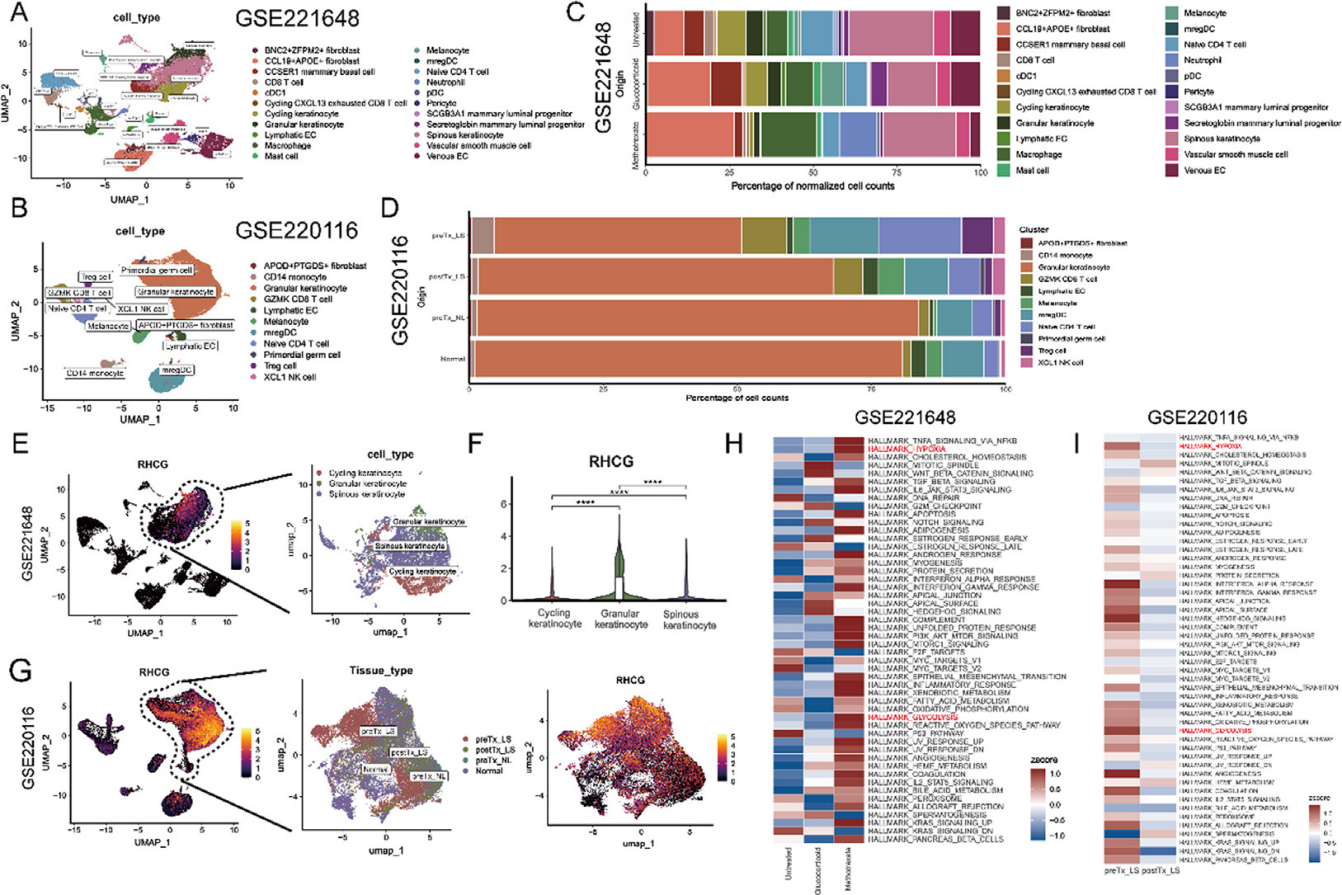

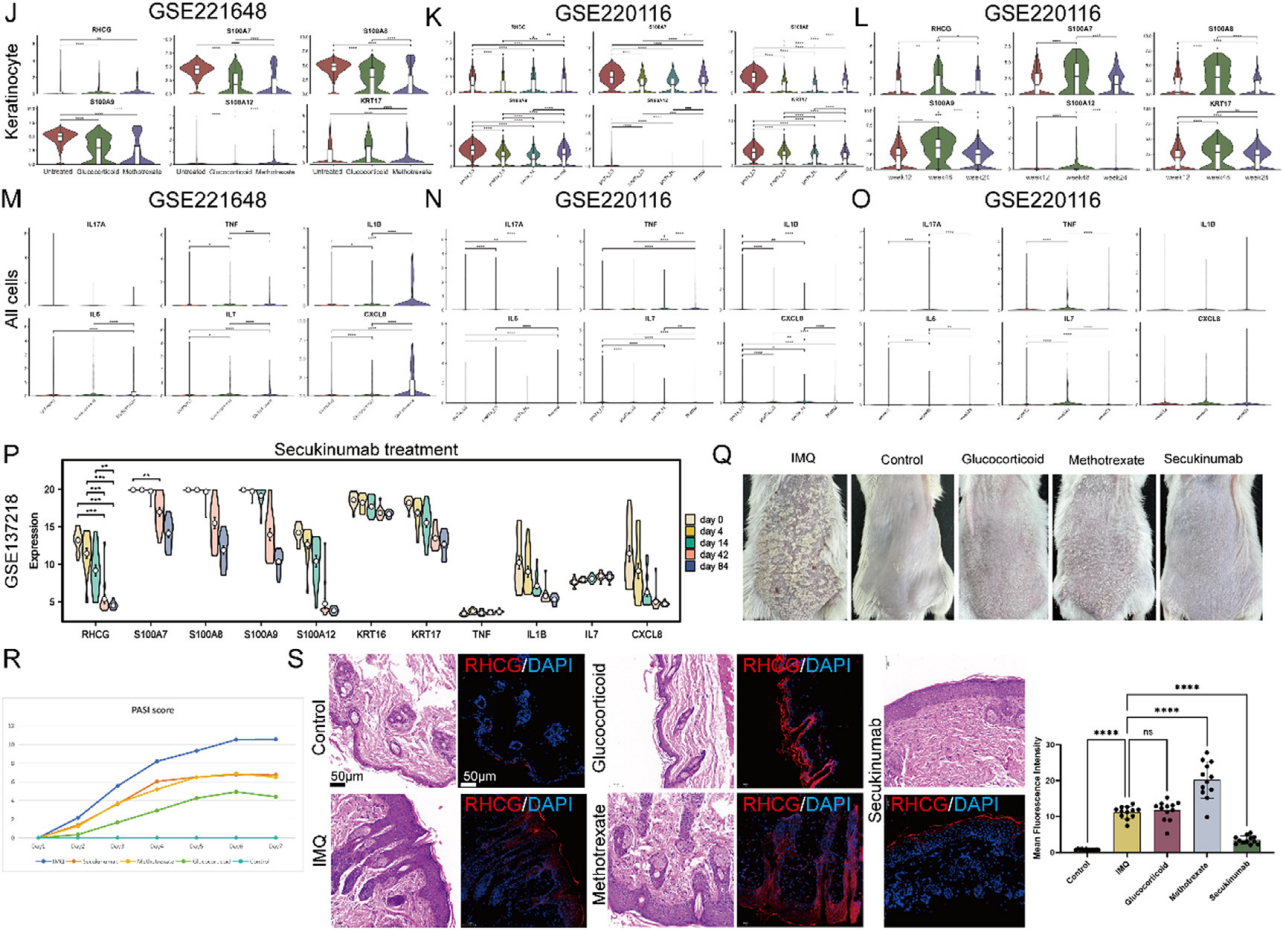
Analysis of RHCG expression and its regulatory pathways in psoriasis across treatment conditions. (a) UMAP projection of single-cell RNA sequencing data from the GSE221648 dataset, displaying distinct cell populations in psoriatic lesions. Each dot represents an individual cell, color-coded according to cell type based on gene expression profiles. See also Fig. S7A. (b) UMAP projection of single-cell RNA sequencing data from GSE220116, showing cell type distributions in both psoriatic and healthy control skin samples. See also Fig. S7B. (c) Bar plot illustrating the percentage of normalized cell counts for each cell type in the GSE221648 across different treatment conditions, including methotrexate, glucocorticoid, and untreated groups. (d) Bar plot showing the relative proportions of major cell types across different skin sample origins in GSE221648, including preTx_LS (pretreatment lesional skin), postTx_LS (post-treatment lesional skin), preTx_NL (pretreatment non-lesional skin), and Normal (healthy control skin). (e) Spatial feature plot from GSE221648 highlighting the expression levels of *RHCG* across different keratinocyte subtypes. The plot displays a gradient color scale, where areas with higher expression levels of *RHCG* are represented by warmer colors, indicating robust expression in specific keratinocyte populations. Notably, keratinocytes within the highlighted regions exhibit significantly elevated *RHCG* expression, suggesting their potential role in inflammatory responses associated with psoriasis. This spatial distribution underscores the relationship between keratinocyte activation and the pathological features observed in psoriatic lesions, as evaluated by the intensity of color representation on the UMAP. (f) Violin plots showing the expression of *RHCG* in different keratinocyte subtypes, with statistical significance indicated. (g) UMAP projections from the GSE220116 dataset, depicting spatial characteristics of keratinocyte populations in psoriatic skin samples. The left panel highlights *RHCG* expression levels, with the color gradient indicating varying expression intensities; warmer colors correspond to higher levels of *RHCG* expression, particularly evident in the outlined region. This suggests a focal accumulation of RHCG within specific keratinocyte populations in lesional skin. The middle panel displays the tissue type distribution, categorizing cells into preTx_LS, postTx_LS, preTx_NL, and normal skin. Each tissue type is color-coded for clarity. The right panel again illustrates *RHCG* expression levels across the spatial landscape of the UMAP, reinforcing the correlation between *RHCG* expression and the defined tissue types, particularly emphasizing the dysregulation observed in psoriatic conditions. Pathway enrichment analysis of hallmark gene sets in GSE221648 (h) and GSE220116 (i). Combined heatmaps reveal hallmark pathway enrichment across treatment conditions in the GSE221648 and GSE220116. Notably, the pathways HALLMARK_HYPOXIA and HALLMARK_GLYCOLYSIS (highlighted in red) show significant upregulation, underscoring the critical role of hypoxic conditions and enhanced glycolytic activity in psoriatic lesions. (j) Violin plots in the GSE221648 showing expression of keratinocyte-related genes (*RHCG*, *S100A7*, *S100A12*, and *KRT17*) across untreated, glucocorticoid, and methotrexate-treated samples. (k) and (l) Expression of keratinocyte-related genes across treatment conditions in GSE220116. Violin plots depict the expression levels of keratinocyte-related genes *RHCG*, *S100A7*, *S100A8*, *S100A12*, *KRT17*, and *S100A9* in the GSE220116. Expression is assessed across different treatment conditions, including preTx_LS, postTx_LS, and normal skin, as well as at various time points (weeks 12, 24, and 48) following treatment. Significant differences in gene expression are indicated by asterisks, underscoring the impact of treatment on keratinocyte activation and the inflammatory response. (m) Expression of key inflammatory cytokines across treatment conditions in GSE221648. Violin plots depict the expression levels of inflammatory cytokines *IL17A*, *TNF*, *IL1B*, *IL6*, *IL7*, and *CXCL8* in all cell types across different treatment groups: Untreated, Glucocorticoid, and Methotrexate. (n) and (o) Violin plots display the expression levels of inflammatory cytokines *IL17A*, *TNF*, *IL1B*, *IL6*, *IL7*, and *CXCL8* across treatment conditions in the GSE220116. Panel (n) shows expression in preTx_LS, postTx_LS, and normal skin, with significant elevations in lesional skin highlighting the inflammatory state of psoriasis. (o) presents cytokine expression at various time points (weeks 12, 24, and 48) following treatment, revealing substantial reductions in *IL17A*, *TNF*, and *IL6* levels over time, particularly in treated samples. (p) Violin plots illustrating the expression levels of keratinocyte and inflammatory genes (*RHCG*, *S100A7*, *S100A8*, *S100A9*, *S100A12*, *KRT16*, *KRT17*, *TNF*, *IL1B*, *IL7*, and CXCL8) following secukinumab treatment over time (days 0, 4, 12, 14, and 84). Significant differences in gene expression are indicated by asterisks, demonstrating the impact of secukinumab on regulating these key inflammatory and keratinocyte-related genes throughout the treatment timeline. (q) Representative images showing the skin of mice in the IMQ-induced psoriasis model, comparing the effects of secukinumab, glucocorticoids, methotrexate, and control treatments. The images depict the severity of psoriasis-like lesions, with secukinumab treatment leading to a marked improvement in skin condition, illustrating its therapeutic potential in reducing psoriatic inflammation and lesions compared to untreated and other treatment groups. See also Fig. S8. (r) Line graph illustrating the Psoriasis Area and Severity Index (PASI) scores over a 7-day period for mice subjected to IMQ treatment, with scores represented for various treatment groups: IMQ, secukinumab, methotrexate, glucocorticoid, and control. The graph shows that secukinumab significantly reduces PASI scores compared to control and other treatments, indicating effective alleviation of psoriatic symptoms. (s) Histological examination of skin samples from the IMQ-induced psoriasis model, featuring H&E staining. Representative images display the skin structure in the control, glucocorticoid, methotrexate, and secukinumab groups, alongside an IMQ group. Fluorescent staining for RHCG (red) and DAPI (blue) highlights keratinocyte responses. The accompanying bar graph quantifies the mean fluorescence intensity of RHCG expression. Scale bars indicate 50 *μ*m. Experiments were carried out in triplicate, and the resulting data were presented as mean ± SD. ns represented no significance; ^****^P < 0.0001, by ANOVA.

Traditional psoriasis treatment typically involves topical agents such as corticosteroids for initial management, aiming to alleviate symptoms.^42,43^ For more severe cases, systemic therapies like methotrexate and cyclosporine are employed to modulate the immune response.^44^ Recent advancements in targeted therapies, particularly biologics such as TNF-α inhibitors, IL-17 inhibitors (e.g., secukinumab), and IL-23 inhibitors, have significantly improved clinical outcomes with a more favorable safety profile.^45^ To explore whether RHCG-related signaling is differentially regulated by these therapies, we analyzed scRNA-seq datasets from Frost *et al.* and Kim *et al.* (GSE221648 and GSE220116). The processing workflow of these scRNA-seq datasets is presented in Figs. 6(a) and 6(b) and Figs. S7A and 7B, with uniform manifold approximation and projection (UMAP) and heatmaps illustrating the marker genes for each cell type. Cellular fractions under different treatments are depicted in Figs. 6(c) and 6(d), and RHCG expression levels are presented in Figs. 6(e)–6(g). Violin plots indicated the upregulated RHCG levels in KCs, particularly in granular KCs [Fig. 6(f)], reaffirming our previous findings. We next assessed functional enrichment using Hallmark gene sets via the gene set variation analysis (GSVA) method to characterize the functional status of cells across different treatment groups. Interestingly, in GSE221648, treatment with glucocorticoids and methotrexate resulted in increased activity of hallmark signaling pathways, particularly those involving hypoxia and glycolysis [Fig. 6(h)]. In contrast, secukinumab exhibited a strong inhibitory effect on hallmark pathways, significantly downregulating nearly all gene sets [Fig. 6(i)]. Considering the established role of RHCG in the hypoxic phenotype, we compared the levels of RHCG-related signaling molecules across the treatment groups. Surprisingly, while glucocorticoids and methotrexate decreased the expression of some pathogenic factors, such as *S100A7*, *S100A8*, and *S100A9*, they paradoxically increased the expression of both *RHCG* and S*100A12* [Fig. 6(j)]. In contrast, secukinumab not only reduced the expression of S100A coding genes and *KRT17* but also significantly inhibited *RHCG* expression [Fig. 6(k)]. We further assessed RHCG-related signaling molecules at different time points following secukinumab treatment. After extended treatment (48 weeks), an increase in the expression of *RHCG* and related molecules was observed, suggesting a potential mechanism underlying drug tolerance [Fig. 6(l)]. We also examined the impact of different treatments on overall inflammatory factor expression in psoriatic tissues, as shown in Figs. 6(m)–6(o). Notably, secukinumab demonstrated superior therapeutic efficacy compared to traditional treatments, but its ability to sustain reduced inflammatory levels diminished with long-term use. Based on these findings, we hypothesized that RHCG-associated signaling could be one of the underlying mechanisms contributing to the superior efficacy and safety profile of secukinumab over conventional drugs. To further investigate this, we included an independent dataset, GSE137218, containing bulk sequencing data collected on days 4, 14, 42, and 84 of secukinumab treatment. Similar to our scRNA-seq findings, the data suggested that secukinumab inhibits *RHCG* expression in a time-dependent manner [Fig. 6(p)]. For experimental verification, we assessed the effect of secukinumab on RHCG-related signaling in the imiquimod (IMQ)-induced psoriasis mouse model [Figs. 6(q) and 6(r) and Fig. S8]. Quantitative analysis using mIF revealed that RHCG expression was significantly induced following IMQ application. Methotrexate treatment further elevated RHCG expression, whereas secukinumab markedly reduced RHCG levels in the skin, nearly returning them to normal control levels (Fig. S6). These results suggest that RHCG is a pathogenic factor in psoriasis and represents a potential downstream target for secukinumab.

### Secukinumab inhibits DC cell activation by suppressing CXCL14–CCR4 signaling

**FIG. 7.**
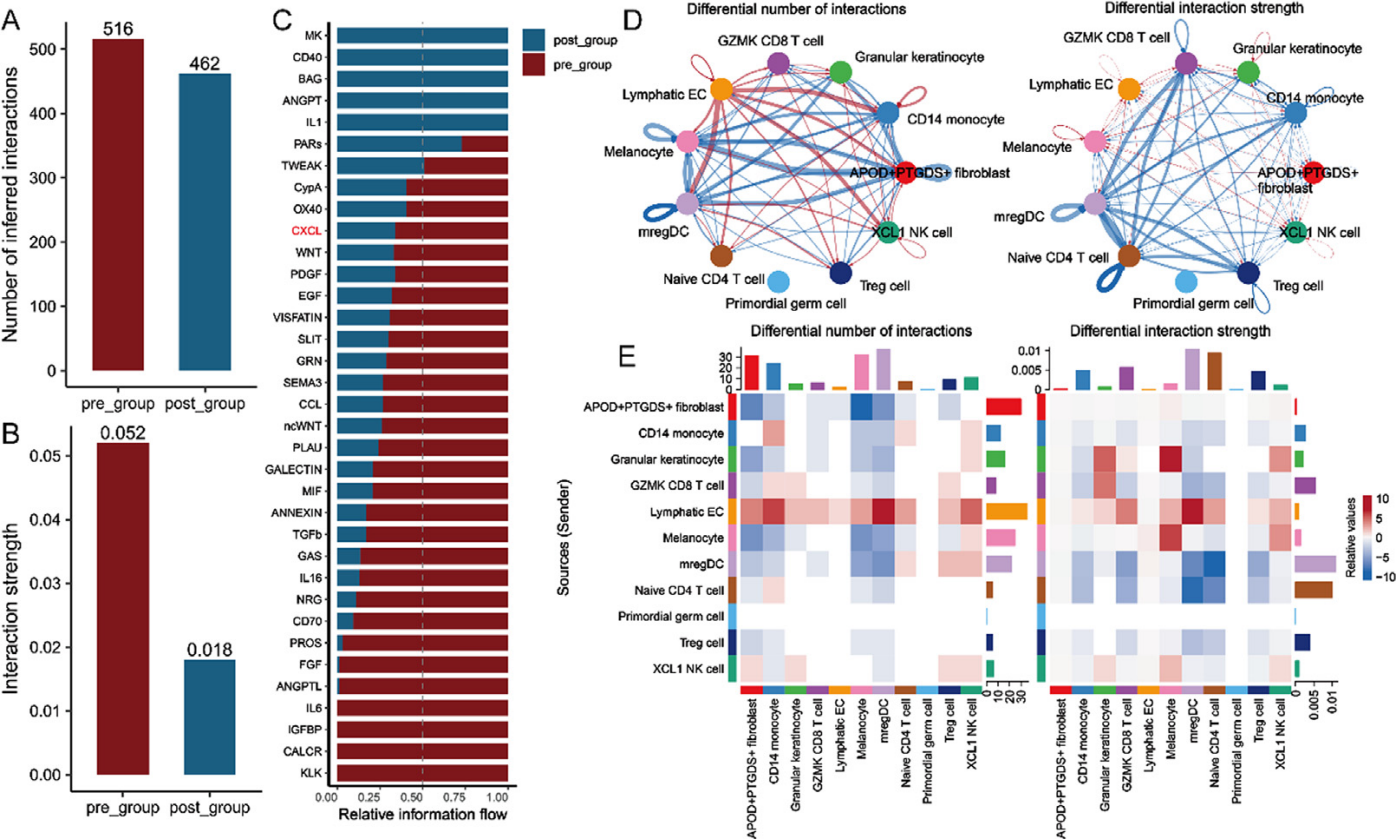

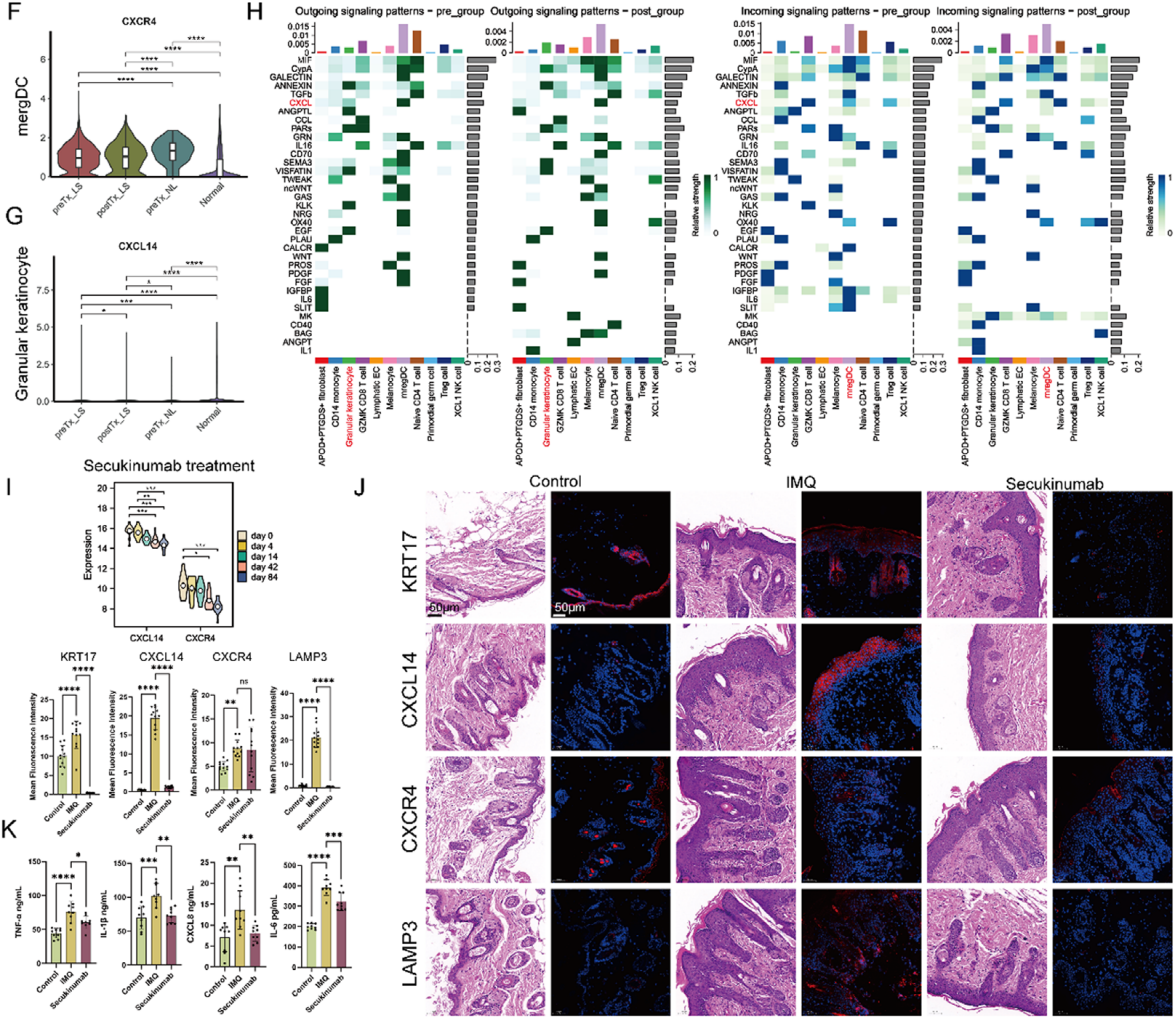
Differential intercellular communication between dendritic cells and keratinocytes under varying treatment conditions in psoriasis. (a) Bar graph displaying the number of inferred interactions in the pre_group (red, 516 interactions) and post_group (blue, 462 interactions). (b) Bar graph illustrating the interaction strength in the pre_group (red, 0.052) compared to the post_group (blue, 0.018). (c) Relative information flow of specific signaling molecules in the pre- and post-treatment groups. The bars represent the strength of interactions for various cytokines and growth factors. (d) Network diagrams illustrate the differential number of interactions (left) and differential interaction strength (right) among immune cell types in the study. (e) Heatmaps depicting the differential number of interactions (left) and differential interaction strength (right) for each immune cell type. The color intensity indicates the level of interaction between cell types, with specific cells demonstrating significant changes in both interaction quantity and strength post-treatment. Violin plots display the expression levels of *CXCR4* in mregDC (regulatory dendritic cells) (f) and *CXCL14* in granular keratinocytes (g) across different sample conditions: preTx_LS, postTx_LS, and preTx_NL (GSE220116). (h) Heatmaps illustrating outgoing and incoming signaling patterns for the pretreatment and post-treatment groups. Each heatmap provides a visual representation of the signaling molecules involved, with color intensity reflecting relative strength. The left heatmaps show outgoing signaling patterns for both pre- and post-group, while the right heatmaps display incoming signaling patterns. (i) Violin plots illustrate the expression levels of *CXCL14* and *CXCR4* across various time points (days 0, 4, 14, 42, and 84) following treatment (GSE137218). (j) Representative images show the expression of KRT17, CXCL14, CXCR4, and LAMP3 in skin samples from the control, IMQ, and secukinumab treatment groups. Each row corresponds to a specific marker, with columns representing different treatment conditions. Scale bars indicate 50 *μ*m. (k) Bar graphs represent the concentrations of inflammatory cytokines: TNF-α, IL-1β, CXCL8, and IL-6 measured in serum samples from control, IMQ, and secukinumab treatment groups. Experiments were carried out in triplicate, and the resulting data were presented as mean ± SD. ns represented as no significance; ^*^P < 0.05; ^**^P < 0.01; ^***^P < 0.001; ^****^P < 0.0001, by ANOVA.

Secukinumab has been demonstrated to effectively diminish the stimulatory impact of inflammatory signals on DCs by specifically targeting and neutralizing interleukin-17A (IL-17A),^46,47^ thereby mitigating DC activation. Given our previous findings that RHCG enhances DC activation, we further investigated whether secukinumab exerts its inhibitory effects on DCs via RHCG-associated pathways. We initially utilized the CellChat tool to conduct a comparative analysis of cellular interactions between secukinumab-treated (post_group) and untreated groups (pre_group). Although no significant difference was observed in the number of receptors or ligands between the secukinumab-treated and untreated control groups, there was a notable reduction in interaction strength in the post-treatment group compared to the pretreatment group [Figs. 7(a) and 7(b)]. Importantly, the levels of CXCL signaling, of particular interest in this context, were significantly suppressed in the secukinumab-treated group [Fig. 7(c)]. Further analysis revealed that both the number and strength of signaling pathways from KCs to monocytes and DCs were significantly diminished in the post-treatment group compared to the pretreatment group [Figs. 7(d) and 7(e)]. Both *CXCR4* and *CXCL14* show significantly elevated expression in all psoriasis-related conditions compared to normal skin. *CXCR4* remains elevated even postTx_LS, suggesting ongoing immune activation, while *CXCL14* expression decreased somewhat, indicating a treatment targeting ligand (rather than the receptor) [Figs. 7(f) and 7(g)]. Heatmap analysis highlighted a significant decrease in CXCL signaling outflow originating from KCs, as well as a corresponding reduction in CXCL signaling inflow to DCs in the post-treatment group [Fig. 7(h)]. These findings were further corroborated by bulk sequencing data [Fig. 7(i)].

To validate the impact of secukinumab on CXCL14–CXCR4 signaling, we performed immunofluorescence analysis of skin sections and an ELISA assay of serum from mouse models. As illustrated in Fig. 7(j), treatment with secukinumab suppressed the activation of DCs (labeled with LAMP3) as well as the aberrant differentiation of KCs (labeled with KRT17) in IMQ-induced psoriasis mice. Additionally, secukinumab treatment reduced the level of CXCL14 while leaving CXCR4 expression unchanged compared to the untreated model group. Secukinumab also led to a decrease in overall levels of inflammation-related factors.

These results suggest that secukinumab inhibits DC activation by downregulating CXCL14–CXCR4 signaling, thereby interfering with the inflammatory crosstalk between KCs and DCs in psoriasis.

### Secukinumab may exert downstream effects by downregulating HIF-1α expression to inhibit RHCG transcription

Based on our findings, it is plausible that secukinumab inhibits DC activation by downregulating RHCG expression, which consequently reduces CXCL14 secretion from KCs. Here, we further investigate the underlying mechanisms. Hypoxia-induced changes in transcription factors, particularly HIF-1α, play a critical role in psoriasis pathogenesis. These changes drive hyperproliferation of KCs, angiogenesis, and immune cell recruitment by modulating glucose metabolism and inflammatory pathways, thus offering potential therapeutic targets for disrupting the psoriatic inflammatory cycle.

**FIG. 8.**
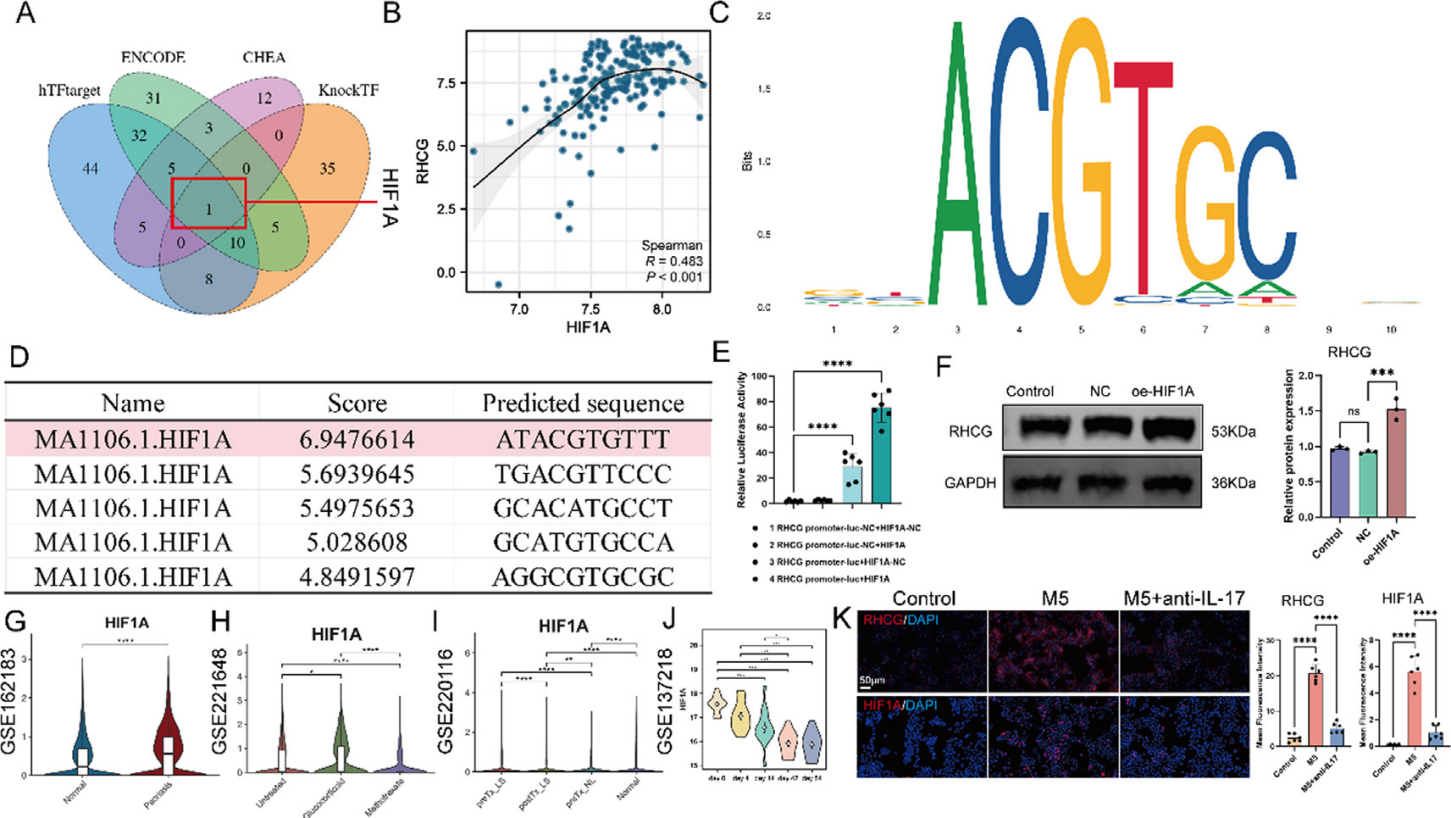

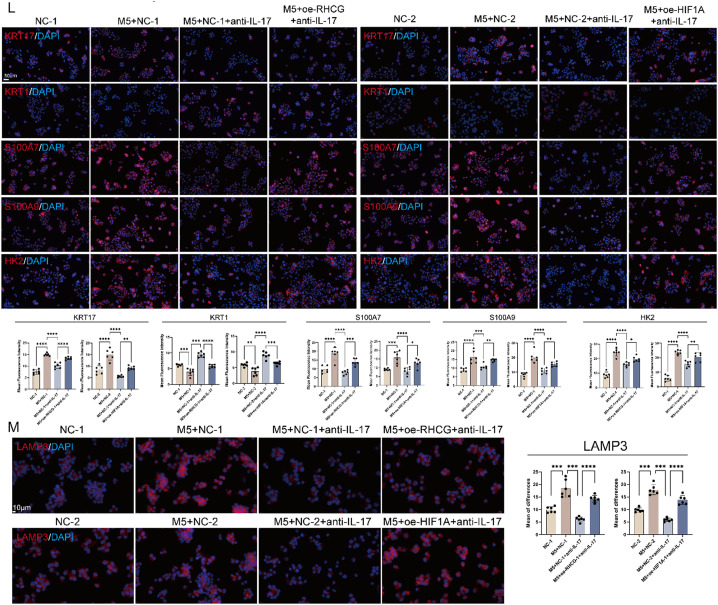
HIF1A-mediated regulation of RHCG and its impact on keratinocyte and dendritic cell activation in psoriasis. (a) Venn diagram illustrating the overlap of transcription factor target genes across multiple databases: hTFtarget, ENCODE, CHEA, and KnockTF. The diagram highlights the unique intersection point labeled 1, which corresponds to *HIF1A*, indicating that it is the only transcription factor common to all datasets. (b) Scatter plot demonstrating the correlation between the expression levels of *HIF1A* and *RHCG*. The Spearman correlation coefficient (R = 0.483) indicates a moderate positive correlation, with a statistically significant p-value (P < 0.001). The fitted line illustrates the trend of increasing RHCG expression with higher levels of HIF1A, suggesting a potential regulatory relationship between these two genes. (c) Sequence logo representing the binding motif for *HIF1A*, illustrating the nucleotide preferences at each position. The height of each letter corresponds to its frequency, indicating the specific binding characteristics of *HIF1A*. (d) Table detailing predicted target sequences for HIF1A, including their associated scores and predicted binding affinities, emphasizing the importance of these sequences in HIF1A-mediated transcriptional regulation. (e) Bar graph showing relative luciferase activity of different RHCG promoter constructs co-transfected with either a negative control (NC) or HIF1A. (f) Western blot analysis confirming increased RHCG and HIF1A protein expression following HIF1A overexpression. The quantification shows significant upregulation of RHCG in the oe-HIF1A group compared to negative control groups (^***^P < 0.001). Violin plots showing expression levels of HIF1A across various datasets (g: GSE162183; h: GSE221648; i: GSE220116) and over time in GSE137218 (j). Statistical significance indicates that *HIF1A* expression is significantly elevated in psoriatic skin compared to normal (^****^P < 0.0001) and varies across treatment conditions and time points. (k) Fluorescence images demonstrating the expression of RHCG and HIF1A in different treatment conditions. Significant differences in mean fluorescence intensity of RHCG and HIF1A are noted, reflecting the impact of IL-17 inhibition on their expression. (l) Representative fluorescence images showing the expression of KRT17, KRT1, S100A7, S100A9, and HK2 in HaCat across various treatment conditions. Each row corresponds to a specific marker, with the DAPI stain used to visualize nuclei (blue) and the markers stained in red. The accompanying bar graphs quantify the mean fluorescence intensity for each marker, indicating significant differences in expression levels across the treatment groups. Scale bars indicate 80 *μ*m. (m) Representative fluorescence images show the expression of LAMP3 in dendritic cells (DC) co-cultured with HaCaT cells under different treatment conditions. LAMP3 is stained in red, while nuclei are visualized with DAPI (blue). Scale bars indicate 10 *μ*m. Experiments were carried out in triplicate, and the resulting data were presented as mean ± SD. ns represented as no significance; ^*^P < 0.05; ^**^P < 0.01; ^***^P < 0.001; ^****^P < 0.0001, by ANOVA.

To explore the regulatory relationship between HIF-1α and RHCG, we searched several transcription factor-target regulatory databases, including hTFtarget, ENCODE, CHEA, and KnockTF. Through intersection analysis, we identified HIF-1α as the sole potential transcription factor regulating RHCG [Fig. 8(a)]. Bulk RNA sequencing data further revealed a statistically significant positive correlation between *HIF1A* and *RHCG* transcript levels [Fig. 8(b)]. To gain insight into the binding mechanism, we used the JASPAR tool to identify an active binding site for HIF-1α within the *RHCG* promoter region [Figs. 8(c) and 8(d)]. Luciferase reporter assays validated that HIF-1α can bind directly to the *RHCG* promoter region [Fig. 8(e)]. Additionally, Western blot analysis demonstrated that upregulation of HIF-1α significantly enhanced RHCG expression in KCs [Fig. 8(f)]. Together, these findings underscore the regulatory role of HIF-1α in modulating RHCG expression.

scRNA-seq data showed that secukinumab is superior to glucocorticoids and methotrexate in ameliorating hypoxic conditions in psoriasis, as evidenced by the downregulation of HIF-1α expression [Figs. 8(g)–8(i)]. Furthermore, secukinumab inhibited HIF-1α expression in a time-dependent manner [Fig. 8(j)]. Prior research established that the M5 cytokine cocktail elicits a psoriasis-like phenotype *in vitro* in cultured KCs, accompanied by increased RHCG expression. To assess the influence of RHCG-associated signaling on the therapeutic efficacy of secukinumab, HaCaT cells were treated with the M5 cytokine cocktail for 24 h, consistent with previous studies.^19^ We observed a concurrent increase in both RHCG and HIF-1α expression following M5 stimulation [Fig. 8(k)].

Immunofluorescence analysis demonstrated that secukinumab effectively suppressed the M5-induced upregulation of psoriasis-related markers, including KRT17, S100A7, S100A9, and HK2, in HaCaT cells [Fig. 8(l)]. Notably, secukinumab also restored the expression of KRT1, a marker of normal keratinocyte differentiation. However, the beneficial effects of secukinumab on inhibiting psoriasis-related markers and restoring KRT1 expression were significantly reduced in the presence of overexpression of RHCG (oe-RHCG) or HIF-1α (oe-HIF1A) [Fig. 8(l)].

To further elucidate the role of RHCG-related signaling in the inhibition of DC activation by secukinumab, we examined the effect of secukinumab on DC activation in a co-culture system. Secukinumab treatment inhibited the elevation of LAMP3 protein, a marker of DC maturation, following M5 treatment in KCs [Fig. 8(m)]. However, this inhibitory effect was reversed by overexpression of RHCG or HIF-1α [Fig. 8(m)]. Together, these observations suggest that the HIF-1α/RHCG axis plays a critical role in the therapeutic effects of secukinumab in psoriasis.

## DISCUSSION

Our study explores the complex role of RHCG in psoriasis, a chronic, immune-mediated skin disorder characterized by abnormal differentiation and proliferation of KCs, immune cell infiltration,^48^ and inflammation.^49^ By leveraging a combination of bioinformatics tools and experimental approaches, we gained significant insights into the mechanisms underlying RHCG's involvement in psoriasis pathogenesis.

First, we observed a strong correlation between RHCG and S100A12, a calcium-binding protein known to be upregulated in inflammatory conditions,^50^ including psoriasis.^51^ This prompted us to further investigate the signaling pathways associated with RHCG. Functional enrichment and spatial transcriptomic (ST RNA-seq) analyses demonstrated that *RHCG* is highly expressed in psoriatic lesions, particularly in KCs. These observations were validated through fluorescence staining and immunohistochemical analysis, which confirmed the high expression levels and co-localization of RHCG and S100A12 in psoriatic tissues.

Moreover, our findings indicated that RHCG expression is associated with hypoxia and glycolysis in psoriasis. The epidermis, where KCs are located, is relatively thin and lacks a direct blood vessel supply, rendering it prone to hypoxic conditions.^52^ In psoriasis, these hypoxic regions extend into the dermis, contributing to chronic inflammation and hyperproliferation. This expansion drives angiogenesis, immune cell infiltration, and glycolytic activity, thereby perpetuating the inflammatory cycle.^26^ Understanding these mechanisms is essential for developing targeted therapies that could interrupt this vicious cycle. Hypoxia-inducible factor (HIF)-1α, which is activated under hypoxic conditions, plays a pivotal role in enhancing glycolytic activity by upregulating key glycolytic enzymes and glucose transporters in KCs.^53^ Our results showed a positive correlation between RHCG expression, HIF-1α, and glycolysis-related genes, suggesting that *RHCG* may be regulated through hypoxia-related pathways in psoriasis.

We also demonstrated that overexpression of RHCG leads to impaired KC differentiation and an inflammatory phenotype. Through ST analysis, we identified nonrandom expression patterns of specific gene features associated with hypoxia, showing differential expression gradients between differentiated KCs and RHCG/S100A12 in psoriasis samples. Our *in vitro* experiments confirmed that RHCG overexpression significantly promoted the expression of inflammatory markers and glycolytic enzymes, highlighting its role in psoriasis pathogenesis.

In addition to its impact on keratinocytes, our study also revealed a significant correlation between RHCG expression and immune cell infiltration, particularly activated DCs. We demonstrated that RHCG overexpression in KCs promotes DC activation and enhances the secretion of inflammatory factors, such as CXCL14, thereby intensifying the crosstalk between KCs and DCs. These findings suggest that RHCG plays an essential role in amplifying the immune response in psoriasis.

In the context of therapeutic interventions, secukinumab, a fully human monoclonal antibody targeting IL-17A, has been shown to exert an inhibitory effect on HIF-1α expression.^54^ By specifically binding to and neutralizing IL-17A, secukinumab disrupts the inflammatory cascade, thus attenuating downstream effects associated with HIF-1α upregulation, including angiogenesis, metabolic dysregulation, and sustained inflammation.^55–57^ This points to a complex interplay between inflammatory and hypoxic signaling pathways, which secukinumab appears to modulate effectively. Importantly, our study showed that secukinumab downregulates RHCG expression in KCs, suggesting that this effect is mediated through HIF-1α regulation. Secukinumab inhibited both the inflammatory and hypoxic/glycolytic phenotypes associated with psoriasis by modulating the HIF-1α–RHCG axis, thereby disrupting the pathogenic communication between KCs and DCs. While our findings establish that IL-17A upregulates RHCG via HIF-1α activation, the precise intracellular pathways linking IL-17A to HIF-1α remain incompletely characterized. Previous studies suggest NF-κB and STAT3 signaling may mediate this process,^58^ though further investigation is needed to fully elucidate this regulatory mechanism.

These findings suggest a novel metabolic regulatory role for secukinumab in the psoriatic microenvironment. The observed downregulation of glycolytic markers (including HK2) following Secukinumab treatment indicates that beyond its primary function as an IL-17A inhibitor, Secukinumab may normalize the metabolic alterations characteristic of psoriatic lesions. This metabolic reprogramming away from enhanced glycolysis could contribute significantly to resolving the hyperproliferative keratinocyte phenotype. While recent research has begun to recognize the importance of metabolic regulation in inflammatory skin diseases,^59–61^ studies specifically examining Secukinumab's impact on cellular metabolism remain limited.^62^ Our observations point to metabolic normalization as a potentially underappreciated mechanism contributing to Secukinumab's superior efficacy compared to conventional treatments.

Importantly, our spatial transcriptomic analysis revealed that RHCG upregulation is not limited to cutaneous psoriasis but extends to PA lesional tissues. RHCG upregulation in PA suggests shared pathogenic mechanisms with psoriasis. The HIF-1α/RHCG axis could represent a common therapeutic target in both conditions.

Consequently, the regulation of the HIF-1α–RHCG signaling axis by secukinumab represents a pivotal mechanism underlying its beneficial effects in ameliorating the pathophysiological manifestations of psoriasis. These findings suggest that the modulation of RHCG-associated signaling could be one of the key reasons behind secukinumab's superior efficacy and safety compared to conventional therapies, such as glucocorticoids and methotrexate.

Despite our comprehensive approach, several limitations warrant consideration. The HaCaT cell line, while valuable, may not fully reflect primary keratinocyte behavior in psoriatic lesions. Similarly, the IMQ-induced mouse model cannot perfectly recapitulate the complex, chronic nature of human psoriasis. Our spatial transcriptomic and single-cell analyses, though powerful, have technical constraints in capturing the complete spectrum of cellular dynamics within psoriatic microenvironments. Furthermore, our focus on the HIF-1α/RHCG axis may overlook parallel pathways contributing to disease pathogenesis. Future studies using primary cultures, humanized models, and broader pathway analyses would strengthen these findings.

## CONCLUSION

Hypoxia-induced RHCG plays a critical role in the development and progression of psoriasis, contributing to abnormal keratinocyte differentiation, enhanced inflammatory signaling, and immune cell activation. Our findings indicate that RHCG is a pivotal mediator of the hypoxia-driven pathogenic mechanisms underlying psoriasis. Importantly, the therapeutic effects of secukinumab appear to involve the downregulation of the HIF-1α–RHCG axis, which not only inhibits keratinocyte hyperproliferation and differentiation defects but also disrupts the pro-inflammatory crosstalk between keratinocytes and dendritic cells. Thus, targeting RHCG represents a novel mechanism through which secukinumab exerts its efficacy, highlighting a promising avenue for improving targeted therapeutic strategies in psoriasis management.

## METHODS

A comprehensive inventory of the chemical compounds and antibodies utilized in this study is provided in the supplementary material (Table S1). The concentrations of the antibodies were established according to the manufacturers' recommendations or based on findings from prior research. Additionally, the supplementary materials contain detailed information regarding supplementary figures.

### Origins of public datasets

Bulk sequencing data: The bulk sequencing data utilized in this study were obtained from our prior research.^18^ Specifically, we merged the GSE13355, GSE30999,^63^ and GSE14905^64^ datasets and corrected for batch effects. As this study is a continuation of our previous work, no further processing was conducted on the merged dataset. Additionally, we incorporated the secukinumab treatment dataset, GSE137218,^65^ into our analysis.

Single-cell RNA sequencing (scRNA-seq) data: The scRNA-seq dataset GSE162183,^66^ which focuses on psoriasis, was obtained from the Gene Expression Omnibus (GEO) database.^67^ This dataset comprises scRNA-seq profiles of full-thickness skin samples from the lesional areas of three patients and corresponding samples from three healthy donors. GSE162183 is primarily utilized to investigate the biological functions associated with RHCG. Additionally, the glucocorticoid- and methotrexate-treatment dataset (GSE221648)^68^ and the secukinumab-treatment dataset (GSE220116)^69^ were similarly downloaded for further analysis.

Spatial transcriptome (ST) data: ST data were retrieved and downloaded from the CROST portal^70^ and the following keywords were used as a search strategy: psoriasis, psoriatic arthritis. A total of 16 ST samples were included in this study, including 4 psoriatic skins and 4 associated normal controls, and 4 psoriatic arthritic skins and 4 associated normal controls.

### scRNA-seq data processing

The scRNA-seq data were processed using the Seurat V5 framework. We utilized the uniform manifold approximation and projection (UMAP) coordinates and cell annotation information obtained from the Deeply Integrated human Single-Cell Omics (DISCO) database^71^ without additional processing. All visualizations and enrichment analyses were performed with the “SeuratExtend” package.^72^ For the analysis of intercellular communication, we employed ligand–receptor interaction data from the “CellChat”^73^ and “CellCall” packages.^74^ Additionally, pseudotime trajectory analysis was conducted using the “SCORPIUS” package.^75^

### ST data processing

ST data were processed using the “SPATA2” package,^76^ which focuses on inferring histology-associated gene expression gradients. In this study, we filtered and outlined the spatial extent of data spots based on their gene set variation analysis (GSVA)-based hypoxia scores.^77^ Additionally, visualizations related to the ST data were generated using this package. For cell type identification, we utilized the deconvolution results provided by the CROST platform.

### Hematoxylin/eosin (HE) staining

The histopathological assessment of psoriasis tissues was conducted using hematoxylin and eosin (HE) staining. Skin samples from psoriatic lesions were fixed in 10% formaldehyde for 24 h to preserve tissue architecture. Following fixation, the samples underwent dehydration through a gradient of ethanol, transitioning from 70% to 100% ethanol to effectively remove water content. After dehydration, the tissues were embedded in paraffin, and sections of 4 *μ*m thickness were prepared using a microtome. The paraffin sections were deparaffinized by immersion in xylene, followed by rehydration through a descending series of ethanol concentrations (100%, 95%, 70%, and 50%). The sections were then stained with hematoxylin for 5–10 min to visualize the nuclei, followed by eosin staining for 1–2 min to highlight cytoplasmic details. After staining, the sections were mounted with a mounting medium and covered with a coverslip. The stained sections were examined under an upright fluorescent microscope (Nikon, Eclipse Ni-E, Tokyo, Japan) to evaluate histopathological changes associated with psoriasis, including epidermal hyperplasia, inflammatory cell infiltration, and other relevant morphological features.

### Cell lines and culture conditions

The conventional immortalized human keratinocyte cell line HaCaT (Catalog No. CL-0090) was obtained from Wuhan Pu-nuo-sai Life Technology Co. Ltd. (Wuhan, China). HaCaT cells were cultured in Minimum Essential Medium (MEM) supplemented with 10% (v/v) fetal bovine serum (FBS), 1% (w/v) penicillin, and 1% (w/v) streptomycin. Human peripheral blood dendritic cells (immature DCs, Catalog No. CP-H179A) were also sourced from Pu-nuo-sai Life Technology Co. Ltd. These dendritic cells were maintained in Basal Medium supplemented with 10% (v/v) FBS, 1% (w/v) penicillin, 1% (w/v) streptomycin, and growth factors specific for dendritic cells, collectively referred to as Complete Medium for human peripheral blood dendritic cells. All cell lines were incubated at 37 °C in a humidified atmosphere containing 5% CO_2_. To authenticate the cell lines used in this study, short tandem repeat (STR) profiling was performed, ensuring their identity and integrity. Mycoplasma contamination was assessed using standard detection assays, confirming the absence of contamination in the cultured cells. To evaluate the effects of hypoxia on the HaCaT, experimental groups were incubated under hypoxic conditions in a tri-gas incubator, maintaining environments of 0.5% O_2_ (5% CO_2_, 0.5% O_2_, and 94.5% N_2_) or 1% O_2_ (5% CO_2_, 1% O_2_, and 94% N_2_) for 24 h. Control groups were maintained under normoxic conditions (21% O_2_) to assess the relative effects of hypoxia on cellular responses.

### Lentiviral vector production and transduction

Lentiviral vectors were employed to achieve the overexpression of *RHCG* and *HIF1A* in HaCaT cells. The lentiviral constructs were designed, synthesized, and produced by GeneChem Corporation. Transduction of HaCaT cells was conducted according to the manufacturer's protocols. For transduction, HaCaT cells were incubated with recombinant lentiviral particles in the presence of 2 *μ*g/ml polybrene, which enhances the efficiency of viral infection. This incubation was carried out for 24 h to facilitate the incorporation of the viral genome into the host cells. Following this period, the media were replaced, and stable cell lines were selected by adding 1.5 *μ*g/ml puromycin to the culture medium. This selection process allowed for the identification of successfully transduced cells expressing the target genes. The transfection procedure was meticulously conducted in accordance with the manufacturer's guidelines to ensure optimal results. The effectiveness of the transduction was validated through western blotting (WB) analysis, confirming the expression of the overexpressed proteins, RHCG and HIF1A, in the transduced HaCaT cells. The protocol demonstrating the highest transduction efficiency was selected for subsequent experiments.

### Immunofluorescence staining of cells and psoriasis tissue

Cells were seeded onto glass slides within 24-well culture plates. Following the indicated treatment protocols, cells were fixed with 4% formaldehyde for 15 min at room temperature. Subsequently, the cells were permeabilized using 0.3% Triton X-100 for 10 min. After permeabilization, the slides were washed three times with phosphate-buffered saline (PBS) to remove excess detergent.

The cells were then incubated with primary antibodies specific to the target proteins overnight at 4 °C to ensure adequate binding. After primary antibody incubation, the slides were washed again with PBS and subsequently stained with appropriate fluorescently labeled secondary antibodies for 1 h at room temperature. The nuclei were counterstained with 4,6-diamidino-2-phenylindole (DAPI) for 5 min. Immunofluorescence images were acquired using a fluorescence microscope (Nikon, DS-Qi1MC, Tokyo, Japan). For a multicolor immunofluorescence assessment of psoriasis tissue, a tyramide signal amplification (TSA) system was utilized. The sliced psoriasis specimens were dewaxed and rehydrated through a series of ethanol washes. Endogenous peroxidase activity was quenched by treating the sections with 3% hydrogen peroxide (H_2_O_2_) for 30 min. Heat-induced epitope retrieval (HIER) was performed to expose antigen sites, followed by blocking with 3% bovine serum albumin (BSA) for 1 h to inhibit nonspecific interactions. The sections were then labeled with primary antibodies overnight at 4 °C. After washing, the sections were incubated with horseradish peroxidase (HRP)-conjugated anti-rabbit secondary antibodies for 1 h. Subsequently, fluorescent tyramide was applied to amplify the signal, followed by another round of HIER treatment, BSA blocking, and antibody staining. Finally, the nuclei were counterstained with DAPI, and the sections were visualized under a fluorescence microscope (Nikon, DS-Qi1MC, Tokyo, Japan).

### Luciferase reporter assay

The luciferase reporter assay was conducted to assess the transcriptional activity of target promoters, utilizing the Dual-Luciferase Reporter Assay System (Promega). Briefly, HaCaT cells were co-transfected with a firefly luciferase reporter construct containing the promoter region of interest and a Renilla luciferase plasmid as an internal control for normalization. Transfections were carried out using Lipofectamine 3000 in accordance with the manufacturer's protocol. After 24 h of transfection, cell lysates were prepared, and luciferase activities were measured using a luminometer (BioTek, Winooski, VT).

### Western blotting (WB)

The western blotting procedure was conducted as previously described.^78^ Proteins were extracted from lysed cells using radioimmunoprecipitation assay (RIPA) buffer, supplemented with protease inhibitors to prevent protein degradation. Protein concentrations were determined using the Bradford assay to ensure equal loading of samples. Subsequently, sodium dodecyl sulfate–polyacrylamide gel electrophoresis (SDS–PAGE) was performed using a 10% polyacrylamide gel, loading 20 *μ*g of each protein sample. After electrophoresis, proteins were transferred onto a polyvinylidene fluoride (PVDF) membrane using a semi-dry transfer apparatus. The PVDF membrane was blocked for 1 h at room temperature with a 5% bovine serum albumin (BSA) solution in Tris-buffered saline (TBS) containing 0.05% Tween-20 (TBST) to reduce nonspecific binding. Following blocking, the membrane was incubated with primary antibodies specific to the target proteins overnight at 4 °C. After primary antibody incubation, the membrane was washed three times with TBST to remove unbound antibodies. Secondary antibodies conjugated to infrared dyes (IRDye 680RD) were applied for 1 h at room temperature. The protein bands were visualized using the Licor Odyssey near-infrared imaging system. Band intensities were quantified using the Licor Odyssey software, providing accurate measurement of protein expression levels. β-Actin was used as a loading control to normalize protein expression across samples. Band intensities were then analyzed to determine relative protein levels.

### Establishment of co-culture units

HaCaT cells and dendritic cells (DCs) were co-cultured using a non-contact transwell system with 0.4 *μ*m pores. Two distinct co-culture models were established for this study. In the first model, HaCaT cells were transfected with a control plasmid (NC) and oe-RHCG plasmid. Following transfection, the HaCaT cells were co-cultured with immature DCs. The second model involved the transfection of HaCaT cells with either the control plasmid (NC), overexpressing RHCG (oe-RHCG), or overexpressing HIF1A (oe-HIF1A). These transfected HaCaT cells were then stimulated with M5, a cytokine mixture designed to replicate the pathological conditions seen in psoriasis,^79^ consisting of IL-6 (10 ng/ml), IL-17 (10 ng/ml), TNF-α (10 ng/ml), IFN-γ (10 ng/ml), and TGF-α (10 ng/ml) for 24 h to induce inflammatory responses. After the stimulation period, the M5 mixture was removed, and the cells were treated with anti-IL-17 (secukinumab, 1 *μ*M) for an additional 24 h. Subsequently, HaCaT cells (5 × 10^5^ cells per well) were collected and placed in the upper chamber of a 6-well transwell plate, while DCs (1 × 10^5^ cells per well) were seeded in the lower chamber. The co-culture system was maintained for 24 h to facilitate cell-to-cell interactions.^22^

### Enzyme-linked immunosorbent assay (ELISA)

The levels of cytokines in serum and culture medium supernatants were quantified using ELISA kits for the following cytokines: IL-1β, CXCL1, IL-17, CXCL8, TNF-α, IL-7, and IL-6. For sample preparation, serum or culture medium supernatants were transferred into fresh tubes and centrifuged at 3500 rpm for 10 min at room temperature to remove any cellular debris. The clear supernatants were carefully collected for analysis. ELISA was performed according to the manufacturer's instructions provided with the respective kits, which included the following steps. The wells of a microplate were coated with capture antibodies specific to each cytokine and incubated overnight at 4 °C. After coating, the wells were washed and blocked with a blocking buffer to minimize nonspecific binding. The prepared serum or supernatant samples were added to the wells and incubated for a specified period to allow the cytokines to bind to the capture antibodies. Biotinylated detection antibodies were added, followed by streptavidin–horseradish peroxidase (HRP) for signal amplification. A substrate solution was added to each well, and the enzymatic reaction was allowed to develop.

The optical density (OD) values were measured at 450 nm using a microplate reader (Bio-Tek, ELX800, Winooski, VT). The concentrations of cytokines were determined by comparing the OD values to a standard curve generated from known concentrations of each cytokine provided with the ELISA kits.

### Animal experiment

A total of 20 male BALB/c mice, aged 6–8 weeks, were obtained from Henan Sikebisi Biotechnology Co., Ltd. (License No. SCXK (Yu) 2020-0005) and bred in the Experimental Animal Center of the Affiliated Hospital of Nanjing University of Chinese Medicine. All mice were maintained under specific pathogen-free conditions and allowed to acclimatize for 1 week prior to experimentation. All animal procedures were performed in accordance with the ARRIVE 2.0 guidelines and were approved by the Ethics Committee of Jiangsu Provincial Hospital of Traditional Chinese Medicine (Ethics No. 2024NL-KS148).

The 20 mice were randomly divided into five groups: model group (IMQ), control group, secukinumab group, methotrexate group, and glucocorticoid group. For the establishment of the IMQ-induced psoriasis model, the backs of all mice were exposed over an area of approximately 2 × 3 cm. The control group received appropriate vaseline treatment, while the other groups received a topical application of 62.5 mg imiquimod (IMQ) cream daily for a duration of 7 days. Secukinumab was diluted from the stock solution (150 mg/ml) with 0.9% saline to achieve a working concentration of 1.5 mg/ml. Methotrexate: Four tablets of Methotrexate (2.5 mg/tablet) were dissolved in 100 ml of distilled water to create a 0.1 mg/ml methotrexate solution. Glucocorticoid group: Mice in the glucocorticoid group were treated with 200 mg of mometasone furoate cream topically. Mice in the model group received a saline solution, while those in the glucocorticoid group received 200 mg of mometasone furoate cream. The methotrexate group was administered 0.2 ml of the methotrexate solution (1 mg/kg/day) via oral gavage. The secukinumab group received 0.3 ml of diluted secukinumab (1.5 mg/ml) intradermally on days 2 and 6 of the experiment. Assessment of psoriasis severity: The severity of psoriatic lesions was evaluated daily using the Psoriasis Area and Severity Index (PASI) score, assessing erythema, scaling, and infiltration in the affected areas of the mouse back. After 7 days of treatment, mice were anesthetized with sodium pentobarbital and euthanized by cervical dislocation. Skin and serum samples were collected for subsequent analysis.

## SUPPLEMENTARY MATERIAL

See the supplementary material for the following: comprehensive inventory of the chemical compounds and antibodies utilized in this study (Table S1); co-expression network of psoriasis-related genes (Fig. S1); cell type definition of each cluster in normal and psoriasis samples (Fig. S2); cell type definition of each cluster in normal and psoriatic arthritis samples (Fig. S3); RHCG expression not elevated in the peripheral blood of psoriasis patients (Fig. S4); spatial transcriptome to determine the relationship between RHCG and hypoxia as well as glycolysis (Fig. S5); interaction between keratinocytes (KCs) and dendritic cells (DCs) (Fig. S6); single-cell transcriptome of GSE221648 and GSE220116 (Fig. S7); and photographs of the dorsal skin of all animals used for *in vivo* experiments (Fig. S8).

## Data Availability

The data that support the findings of this study are available from the corresponding authors upon reasonable request.
